# Influence of Chemical Profile on the Antioxidant Capacity of Brazilian Stingless Bee Honey

**DOI:** 10.1021/acsomega.5c01134

**Published:** 2025-05-12

**Authors:** Lucas R. de O. Dias, Bruna M. Damm, Bruno F. Paqueli, Bruno Q. Araújo, Gislane C. Oliveira, Diolina M. Silva, Eustaquio V. R. de Castro, Rafael de Q. Ferreira, Álvaro C. Neto

**Affiliations:** † Chemistry Department, Federal University of Espírito Santo, 29075-910 Vitoria, Espírito Santo, Brazil; ‡ Post-graduate Program of Vegetal Biology, Federal University of Espírito Santo, 29075-910 Vitoria, Espírito Santo, Brazil

## Abstract

Chemical compounds found in stingless bee honey (SBH) may impact their biological properties; therefore, the establishment of quality control and standardization protocol is essential. This study investigated the chemical composition and antioxidant capacity of SBH produced by Melipona species from the Espírito Santo state (Brazil). A comprehensive characterization of SBH was conducted using Fourier transform mid-infrared (FT-MIR) and nuclear magnetic resonance (NMR) spectroscopy combined with chemometrics tools. A principal component analysis (PCA) model based on FT-MIR signals showed the separation betweenMelipona capixaba and Melipona quadrifasciata. Volatile organic compounds (VOCs) were detected by gas chromatography–mass spectrometry (GC–MS), and the more abundant classes of the classes were carboxylic acids, ketones, aldehydes, and alcohols. Moreover, the PCA model based on partial GC–MS chromatograms allows the grouping into M. capixaba and M. quadrifasciata. Simple phenols such as *p*-cresol and allyl guaiacol were also identified in SBH. In addition, total phenolic contents (TPC) and total flavonoid contents (TFC) had selective correlations with total antioxidant capacity (TAC). A high Pearson correlation was observed between TPC and ceric reducing antioxidant capacity (CRAC), although there were low correlations among TAC values. Chemometrics combined with phenolics and antioxidant capacity are comprehensive approaches to determine the identity and quality of SBH.

## Graphical abstract

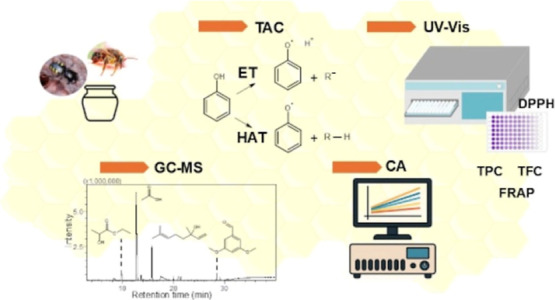

## 1 Introduction

Stingless bees (Meliponini), also known as native bees, are found in tropical and subtropical regions representing more than 500 bee species worldwide.^1^ Although international legislation governs the quality and identity parameters of Apis melliferahoney, there is a need to establish specific standards and regulatory guidelines for stingless bee honey (SBH).^2^ Currently, SBH quality is assessed based on its physicochemical, sensory, and microbiological properties.^3^

In general, honey is a complex mixture of sugars (primarily glucose and fructose), and its chemical composition can vary considerably depending on climatic conditions, stages of maturation, bee species, and flower type as well as processing and storage methods.^4^

SBH is recognized for its characteristic sweetness combined with an acidic taste, a more fluid texture, and slow crystallization. In addition to sugars, enzymes, amino acids, organic acids, minerals, volatile organic compounds, pigments, and pollen grains, SBH contains phenolic compounds that can impact the antioxidant capacity.^3^ Due to the antioxidant, nutritional, and biological properties of SBH, it has attracted economic interest in the past few years.^5,6^

Phenolic compounds are widely distributed in plants from bee pastures.^7^ Several phenolic acids and flavonoids, such as gallic acid, vanillic acid, protocatechuic acid, cinnamic acid derivatives, quercetin, and rutin have been identified in SBH. These molecules contribute to honey’s flavor, aroma, and antioxidant capacity.^8^

Furthermore, several stingless bee species are capable of producing phenolic compounds-rich honey. Therefore, the chemical characterization of this natural product is essential to know its compositional and functional profiles.^9^

In 2020, Kozłowicz and collaborators characterized honey derived from chestnut flowers and honey with dark orange berries, both produced by stingless bees, identifying phenolic compounds using gas chromatography–mass spectrometry (GC–MS) and Fourier transform mid-infrared (FT-MIR) spectroscopy. Several chemical compounds were identified, including 3-phenylpropionic acid and ferulic acid in both samples, while *m*-coumaric acid, benzoic acid, and cinnamic acid were predominantly found in only one honey sample.^10^

The presence of phenolic compounds in honey contributes to its antioxidant responses. In general, the antioxidant capacity can be determined by using spectrophotometric methods such as ferric reducing antioxidant power (FRAP) and DPPH (2,2-diphenyl-1-picrylhydrazyl).^11,12^ Some works have determined the total phenolic and flavonoid contents and verified their correlations. While spectrometric methods are widely employed for the quantification of phenolic compounds and the evaluation of antioxidant capacity, the accuracy of these assessments can be compromised by matrix effects and interfering substances, resulting in weak correlations between measured properties.^13^

Consequently, alternative approaches are necessary to verify the correlation among these physicochemical characteristics. Ceric reducing antioxidant capacity (CRAC) electrochemical assay offers several advantages over spectrophotometric techniques. These include the ability to specifically target the redox state of antioxidants, low cost, potential for miniaturization, rapid analysis when compared to reference methods, insensitivity to light, and the absence of interference from sample coloration.^14^

The objective of the present study is to conduct detailed chemical characterization combined with an evaluation of antioxidant capacity to ensure authenticity and quality control of honey. This knowledge is crucial to addressing existing gaps, encouraging proper regulation, and promoting sustainability in the production of SBH. Consequently, it enables broader acceptance and distribution of this product, benefiting consumers, producers, and biodiversity.

## 2 Materials and Methods

### 2.1 Honey Samples

Twenty honey samples from stingless bees of the genus Melipona were collected in May 2023 from the southwestern mountainous region of Espírito Santo state (Castelo, Domingos Martins, and Venda Nova do Imigrante), considering their importance for meliponiculture and biodiversity conservation efforts (Table 1).

**Table 1:** Stingless Bees Honey Samples and Geographical Settings of the Municipalities from Espírito Santo State (Brazil)

number	code	location	bee species	colony
1	PEMC 01	Domingos Martins—Pedra Azul	Melipona capixaba	1
2	PEMC 02	Domingos Martins—Pedra Azul	Melipona capixaba	2
3	PEMC 01	Domingos Martins—Pedra Azul	Melipona capixaba	3
4	CAMC 01	Domingos Martins—Pedra Azul	Melipona capixaba	1
5	CAMC 02	Domingos Martins—Pedra Azul	Melipona capixaba	2
6	CAMC 03	Domingos Martins—Pedra Azul	Melipona capixaba	3
7	CVMC 01	Domingos Martins—Tijuco Preto	Melipona capixaba	1
8	CVMC 02	Domingos Martins—Tijuco Preto	Melipona capixaba	2
9	CVMC 03	Domingos Martins—Tijuco Preto	Melipona capixaba	3
10	CVMQ 01	Domingos Martins—Tijuco Preto	Melipona quadrifasciata	1
11	CVMQ 02	Domingos Martins—Tijuco Preto	Melipona quadrifasciata	2
12	CVMQ 03	Domingos Martins—Tijuco Preto	Melipona quadrifasciata	3
13	FGMC 01	Castelo	Melipona capixaba	1
14	FGMC 02	Castelo	Melipona capixaba	2
15	FGMC 03	Castelo	Melipona capixaba	3
16	FGMQ 01	Castelo	Melipona quadrifasciata	1
17	DBMC 01	Venda Nova do Imigrante	Melipona capixaba	1
18	DBMC 02	Venda Nova do Imigrante	Melipona capixaba	2
19	DBMC 03	Venda Nova do Imigrante	Melipona capixaba	3
20	VDIMC 01	Venda Nova do Imigrante	Melipona capixaba	1

SBH was exclusively obtained from meliponaries located in the natural habitats of the species, excluding those situated in urban areas, regions with intensive agriculture, or landscapes heavily impacted by anthropogenic activities. Collections were conducted in colonies that had not been artificially fed. Approximately 100 g of samples were collected using sterile syringes, stored in a plastic container, and kept under refrigeration at a temperature of 7 °C until analysis.

It is widely recognized that honey crystallization is related to the proportions of fructose and glucose.^15^ Thus, samples exhibiting this characteristic were subjected to a decrystallization process in a thermostatic water bath (Fisatom, Brazil), maintaining the temperature at approximately 40 °C until the honey decrystallizes. Analyses of each SBH sample were performed in triplicate.

### 2.2 Reagents and Solutions

All aqueous solutions used in the experiments were prepared with ultrapure water (resistivity of 18 MΩ cm, 25 °C) obtained through a reverse osmosis purification system (Sartorius arium mini, Germany). The reagents employed included cerium­(IV) sulfate tetrahydrate (Ce­(SO_4_)_2_·4H_2_O, 98%, Sigma-Aldrich), sulfuric acid (H_2_SO_4_, 98%, Vetec), Trolox (C_14_H_18_O_4_, 97%, Sigma-Aldrich), absolute ethanol (C_2_H_5_OH, 99.8%, Vetec), gallic acid (C_6_H_2_(OH)_3_COOH, 97.5%, Sigma-Aldrich), aluminum chloride (AlCl_3_, 99.5%, Vetec), Folin-Ciocalteu reagent (Sigma-Aldrich), methanol (CH_3_OH, 99.8% Neon), isopropyl alcohol (C_3_H_8_O, 99.5% Neon), and deuterated water (D_2_O, 99.9%, Sigma-Aldrich).

### 2.3 Fourier Transform Mid-infrared (FT-MIR) Analyses

For physicochemical characterization using FT-MIR, a drop of SBH was directly applied in the attenuated total reflectance (ATR) module (Cary 630, Agilent Technologies Inc., USA). FT-MIR spectra were acquired from 4000 to 650 cm^–1^ with 32 scans and a resolution of 4 cm. ATR module was cleaned with analytical-grade isopropyl alcohol for each sample.^16^

### 2.4 Nuclear Magnetic Resonance (NMR) Analyses

SBH samples (20.0 mg) were dissolved in D_2_O. ^1^H NMR spectra were obtained on a 400 MHz spectrometer (VNMRS 400 model, Varian, USA) operating at a magnetic field of 9.4 T, using a probe of 5 mm 1H/X/D Broadband. All spectra were processed using MestReNova software.^17^

### 2.5 Identification of Volatile Organic Compounds by Static Headspace Sampling and Gas Chromatography–Mass Spectrometry (SHS-GC-MS)

The volatile fraction of SBH was analyzed using a GC-MS QP2010 Ultra system (Shimadzu Corporation, Japan) equipped with a static headspace sampler (SHS) and an OV-WAX capillary column (30 m length, 0.25 mm internal diameter, and 0.25 μm film thickness). A 20 mL headspace vial containing 2 g of the sample was incubated in an SHS oven at 95 °C for 15 min. Subsequently, 1 mL of the volatile fraction was collected using a 2.5 mL syringe (Hamilton, USA) and injected in splitless mode (0.45 min) at an injector temperature of 240 °C. The oven temperature program had an initial temperature of 40 °C for 2 min, ramp of 4 °C min^–1^ to 120 °C, and a heating rate of 11 °C min^–1^ to 230 °C, where it was held for 8 min. Helium (99.999%, White Martins, Brazil) was used as the carrier gas at a flow rate of 1.5 mL min^–1^. The mass spectrometer operated in scan mode over a mass range of 35–300 Da, with an ion source temperature of 230 °C, an interface temperature of 220 °C, and a solvent cutoff time of 2.5 min. Chromatographic data were processed using LabSolutions GC–MS Solutions software, version 4.20 (Shimadzu Corporation, Japan). Volatile compound identification was performed by comparing the experimental mass spectra with those in the NIST11 and WILEY7 libraries. Results were expressed as relative area (%) by the integration of peaks in the total ion chromatogram (TIC).^18^ Retention index (RI) was calculated using the equation RI = 100 (*n*) + 100 (*m* – *n*) (*t*_R_ *i* – *t*_R_ *n*)/(*t*_R_ *m* – *t*_R_ *n*), where *m* and *n* are the numbers of carbons of the *n*-alkanes that elute before and after “*i*”, respectively, and “*i*” is a VOC, based on retention time (*t*_R_) of a mixture of *n*C_7_ to *n*C_40_ alkanes (Supelco Analytical).

### 2.6 CRAC Assay

An aliquot of 1.0 g of honey was used to prepare a 50% (w/w) aqueous solution, reducing viscosity and simplifying manipulation. The antioxidant capacity using chronoamperometric measurements was realized using Cottrell’s equation, which expresses current decay as a function of time after applying a potential step to the working electrode (boron-doped diamond, BDD). This allowed monitoring of Ce^4+^ concentration decay after 4 min of reaction with the antioxidant sample. The measurements were performed from the open circuit potential (OCP ∼ 1.20 V) for 5 s, followed by a reduction potential step to a final potential (*E*_2_ ∼ 0.6 V) for 10 s.

To obtain the calibration curve for the Ce^4+^ oxidizing solution, the concentration was varied against the Cottrell slopes obtained from the chronoamperometric assays. Five concentrations (0.2, 0.4, 0.6, 0.8, and 1.0 mol L^–1^) were prepared from the Ce^4+^ 1.0 × 10^–3^ mol L^–1^ stock solution in H_2_SO_4_ 0.5 mol L^–1^. Subsequently, 15 mL of the solution was used to determine the Cottrell slope for each Ce^4+^ concentration. Then, the regression equation from the calibration curve was used to determine the concentration of residual Ce^4+^ species in solution or Ce^3+^ species reduced by the reaction (CRAC value), reflecting the antioxidant’s reduction capacity. This value can also be expressed dimensionless as a Trolox equivalent (TE) dividing the concentration of Ce^3+^ species produced by the reaction with the sample and the Trolox standard, respectively. Accordingly, an aliquot of 100 μL of the samples was added one by one, in triplicate, to 15 mL of the oxidizing solution, and chronoamperometric measurements were performed.^14^

BDD electrode surface was pretreated previously, for each measurement, via anodic treatment (+3.0 V) for 30 s, followed by cathodic treatment (−3.0 V) for 90 s in a H_2_SO_4_ 0.50 mol L^–1^ solution. This cleaning and activation ensured the reliability and reproducibility of the results.^19^

### 2.7 FRAP Assay

The FRAP method was performed according to Rufino et al. (2006), with modifications. For analysis of SBH, a sample solution (100 mg mL^–1^) was prepared using 0.1 mg of SBH and 1 mL of a 50% methanol solution. The FRAP reagent was prepared using 25 mL of acetate buffer (0.3 mol L^–1^), 2.5 mL of 2,4,6-tri­(2-pyridyl)-*s*-triazine (TPTZ, Sigma-Aldrich) (10 mol L^–1^), and 2.5 mL of ferric chloride (Synth) (20 mol L^–1^). In brief, an aliquot of 45 μL of sample solution was added to 1.35 mL of FRAP reagent and 135 μL of distilled water. The mixture was homogenized using a vortex mixer and incubated in a water bath at 37 °C for 30 min. Absorbance readings were taken at 595 nm in a microplate (ELISA, USA) using an Epoch2 microplate reader (Biotek, USA). The FRAP reagent served as the blank, and a standard curve using ferrous sulfate (Sigma-Aldrich, 400–2000 μmol L^–1^) was employed. Results were expressed as μmol FeSO_4_ per 100 g of sample.^20^

### 2.8 DPPH Radical Scavenging Assay

Antioxidant capacity against the DPPH radical was determined according to Pires et al. (2017) with modifications. An aliquot of 100 μL of sample solution was added to 200 μL of 80 μmol L^–1^ DPPH solution (Sigma-Aldrich) in a microplate (ELISA, USA), and the absorbance was taken at 517 nm using an Epoch2 microplate reader (BioTek, USA). A calibration curve of DPPH (0–12.5 μg mL^–1^) and the results were expressed as μg of Trolox per g of sample.^21^

### 2.9 Total Phenolic Contents (TPC)

Phenolic compounds were determined using the Folin–Ciocalteu method, as described by Woisky and Salatino (1998), with modifications. A volume of 25 μL of stock solution was added to 125 μL of Folin–Ciocalteu reagent (Sigma-Aldrich, 1:10 dilution) and 100 μL of 4% sodium carbonate (Dinâmica). The reaction was incubated in the dark for 1 h at room temperature. Absorbance was measured at 740 nm in a microplate (ELISA, USA) by using an Epoch2 microplate reader (BioTek, USA). A calibration curve (5–160 mg mL^–1^) was obtained, and the results were expressed as mg gallic acid equivalents (GAE) per 100 g of sample.^22^

### 2.10 Total Flavonoid Contents (TFC)

TFC was determined according to the method of Dowd (1959), with modifications. An aliquot of 200 μL of a sample solution was mixed with 75 μL of 5% AlCl_3_ (Dinâmica) in methanol and incubated at room temperature for 30 min before measuring the absorbance at 415 nm using a microplate (ELISA, USA) using an Epoch2 microplate reader (BioTek, USA). Flavonoid concentration was calculated using a quercetin calibration curve (0–100 μg L^–1^), and the results were expressed as mg quercetin equivalents (QE) per 100 g of sample.^23^

### 2.11 Chemometric and Statistical Analysis

Chromatography and infrared spectroscopy data were subjected to exploratory analysis using principal component analysis (PCA). Spectroscopic data were organized in matrices of format X_[60,1798]_ with the rows corresponding to the samples and the columns corresponding to an intensity associated with a wavenumber (cm^–1^). Chromatographic data were organized in a table of format X_[57,74000]_, with the rows corresponding to the samples and the columns corresponding to the intensity of retention time (*t*_R_, min). The chromatographic data were also subjected to a selection of variables reducing the matrix to the format X_[57,21003]_, which corresponds to the interval of *t*_R_ from 18.5 to 29.0 min. FT-MIR spectra had baselines adjusted by applying the first derivative and then were centered on the mean. The chromatograms were aligned using the icoshift algorithm and processed by the standard normal variate (SNV) centering the data on the mean. The Pearson correlation chart was developed to evaluate the strength of linear relationships between two variables. In turn, the average values obtained for the TPC, TFC, DPPH, FRAP, and CRAC assays were used for the Pearson correlation coefficient (PCC, *r*). All data analyses were performed using MATLAB software (version R2013A).

Statistical analyses and graphics were performed by using Origin Lab software. A one-way ANOVA test was carried out with a significance level of 0.05.

## 3 Results and Discussion

### 3.1 Spectroscopy Characterization

Figure 1 shows the FT-MIR and NMR spectra for the physicochemical characterization of the Melipona capixaba and Melipona quadrifasciata SBH samples. The FT-MIR spectra exhibited absorption frequency from 3500 to 3100 cm^–1^, which was attributed to O–H stretching from carbohydrates.^24^ The presence of carboxylic acid derivatives, including triacylglycerols and fatty acids, in these samples was attributed to infrared bands around 2935 and 1650 cm^–1^, which are characteristics of the C–H bond with hybridization sp^3^ and the C=O group. In addition, C–O band was observed in absorption frequency around 1020 cm^–1^.^25,26^ As previously highlighted, the chemical groups detected by the FT-MIR spectroscopy data were related to carbohydrates and lipids. These structures were also detected on ^1^H NMR spectra analyses from the SBH samples. The signals at δ 1.0 and 1.2 ppm may be attributed to methyl hydrogens and methylene hydrogens from (−CH_2_−)_*n*_ hydrocarbon chains of fatty acids and triacylglycerols. The signal at δ 2.1 ppm is characteristic of α-methylene hydrogen linked to carbonyl groups from carboxylic acid, such as acetic acid. In addition, two doublets at δ_H_ 4.5 (*J* = 7.8 Hz) and 5.1 (*J* = 3.9 Hz) ppm were attributed to β and α hydrogens (H1) in anomeric carbon from d-glucose, respectively. The signals from δ_H_ 3.0 to 4.0 ppm were attributed to H2, H3, H4, H5, and H6 from α- and β-d-glucose.^27^ Although carbohydrates and lipids, including carboxylic acids, were major compounds found in SBH samples, minor differences were observed in the NMR fingerprinting of M. capixaba and M. quadrifasciata honey.

**Figure 1:**
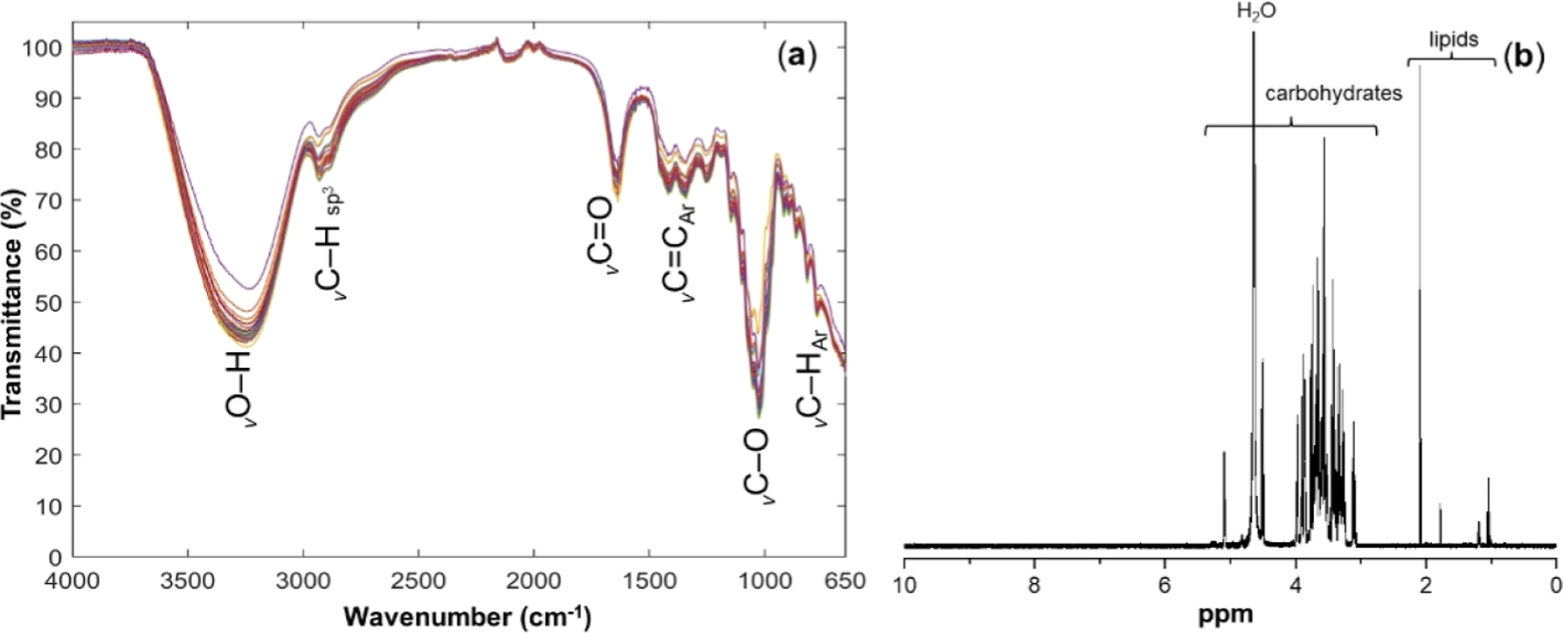
(a) FT-MIR and (b) NMR spectra of stingless bee honey samples. *v*—infrared absorption frequencies (stretching or bending).

PCA plot from the FT-MIR spectral data (Figure 2a) revealed a cumulative variance of 73.87% for the principal components 1 (PC1) and 2 (PC2). A trend of separation was observed along PC1, with the SBH samples separated into two groups. One group located in the positive direction of PC1 (PC1 > 0) included all M. quadrifasciata honey from Domingos Martins—Tijuco Preto and Castelo, and the second group included all M. capixaba honey, which was predominantly located on the negative quadrant of PC1 (PC1 < 0). As observed in Figure 2b, the variables accountable for separation along PC1 were the first-derivative wavenumber on 1025 to 784 and 825 cm^–1^ (PC1 > 0), which are related to the C–O bond of carbohydrates, C–H bending out of plane for aromatic rings of benzene derivatives and phenolic compounds, respectively. Moreover, SBH samples in PC1 < 0 (negative quadrant) were related to 958 cm^–1^, associated with carbohydrates as monosaccharides. The weight variables accountable for the separation of SBH samples on PC2 < 0 (negative quadrant) were 3649 and 1698 cm^–1^, which are characteristic of alcohols and carbonyl group, respectively, while those on PC2 > 0 (positive quadrant) were related to 2861 (*v*C–H *sp* ^3^) and 1565 cm^–1^ (*v*C=C_Ar_).^10^ Thus, M. quadrifasciata honey was grouped based on weight signals of phenolics and aromatic compounds, and M. capixaba honey was grouped based on carbohydrates, lipids, and aromatic compounds. It is possible that the amount and the benzene derivatives-types in M. capixaba and M. quadrifasciata honey can influence the biological activities, especially the total antioxidant capacity.

**Figure 2:**
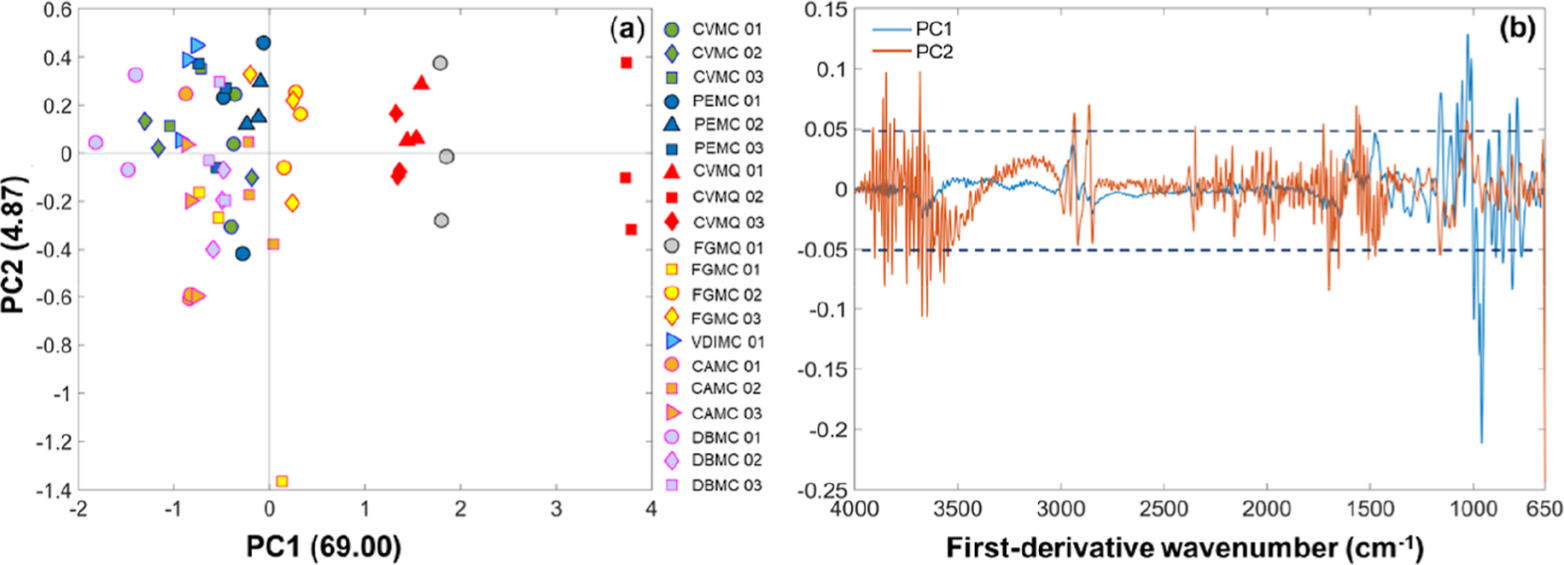
PCA plot showing PC1 *versus* PC2 from FT-MIR data: (a) scores and (b) loadings plots.

### 3.2 Volatile Organic Compounds (VOCs)

Figure 3 shows GC–MS TIC, highlighting the most abundant volatile compounds in the SBH samples from M. capixaba and M. quadrifasciata.

**Figure 3:**
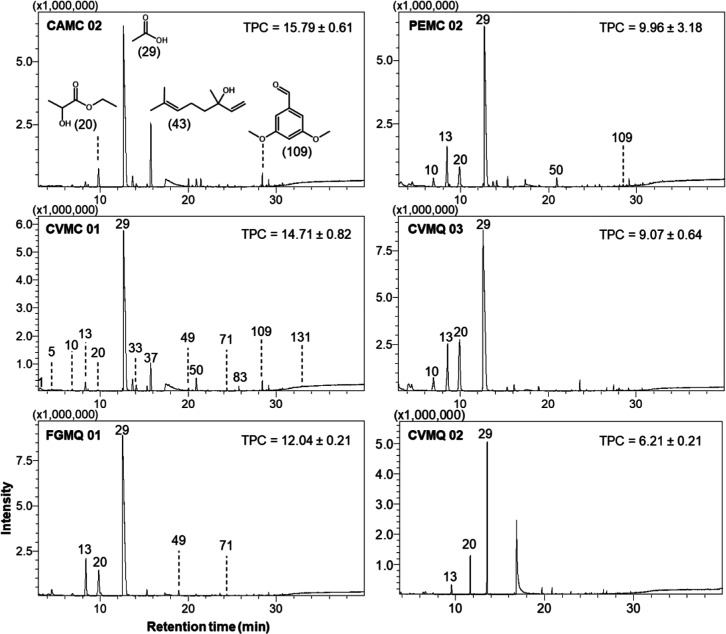
GC–MS TIC for selected M. quadrifasciata and M. capixaba stingless bee honey. TPC was expressed in mg GAE 100 g^–1^ of SBH. (21) 2-Hydroxypropanoic acid ethyl ester. (30) Acetic acid. (44) Hotrienol. (110) 3,5-Dimethoxybenzaldehyde.

The GC–MS analyses allowed the identification of VOCs (Supporting Information, Table S1), which were distributed in eight chemical classes: alcohols, aldehydes, carboxylic acids, esters, ethers, phenols, hydrocarbons, and ketones. As observed, carboxylic acids were the most abundant class in all SBH, with values of 74.34 ± 13.03 and 70.95 ± 5.14% for M. capixaba and M. quadrifasciata, respectively, while phenols and hydrocarbons were minor compounds found in these samples (Figure 4).

**Figure 4:**
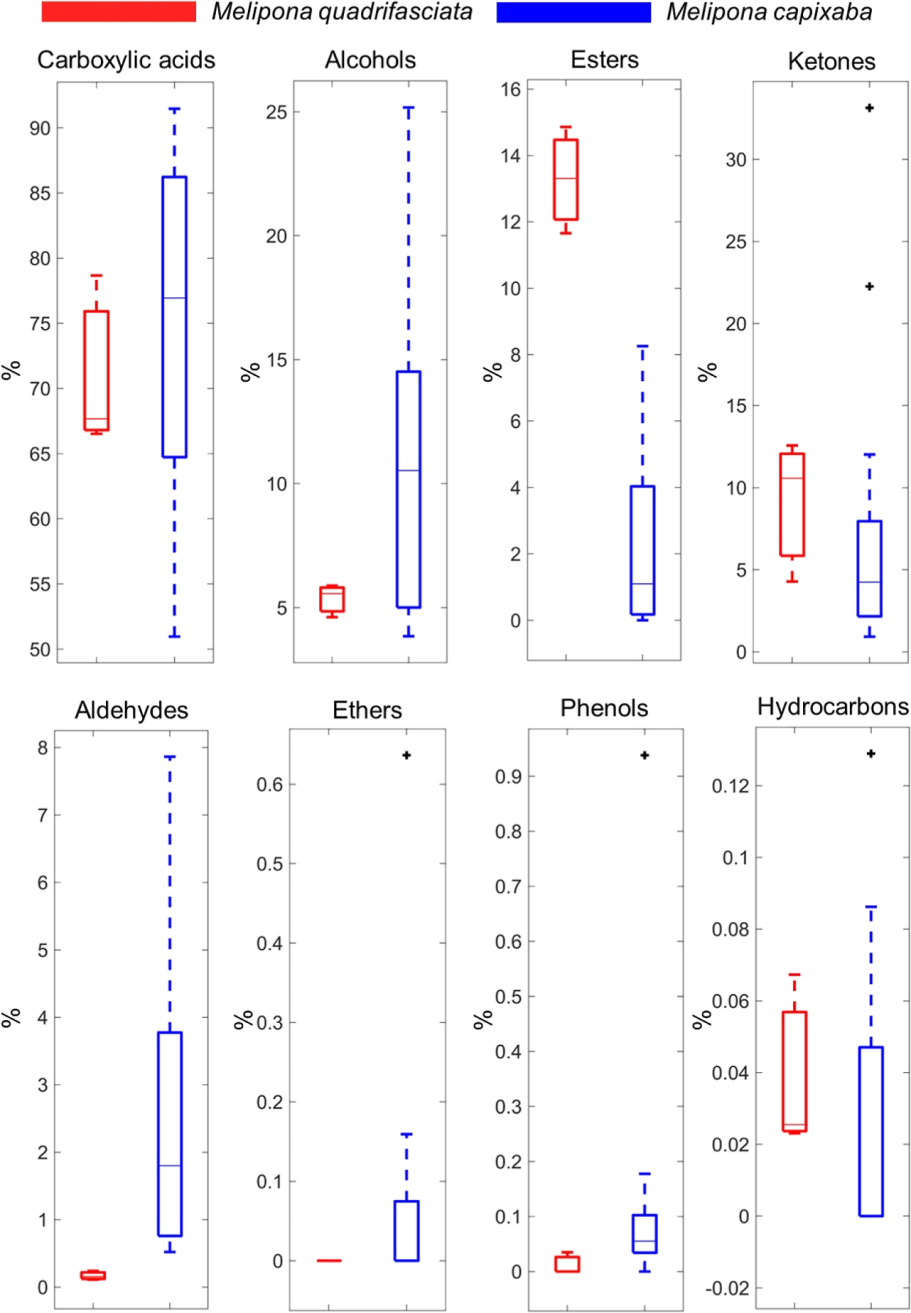
Box plot for chemical classes of M. quadrifasciata (red bar) and M. capixaba (blue bar) stingless bee honey.

#### 3.2.1 Carboxylic Acids

As noted in Figure 4, the main class in all honey samples was carboxylic acid. Thus, acetic acid was the most abundant, with a range from 45.86 ± 0.00 (PEMC 01) to 88.63 ± 4.76% (CVMC 03), as shown in Table S1 (Supporting Information). This compound has the potential to serve as a marker of honey authenticity.^28^ Additionally, the presence of this volatile compound may indicate the presence of honeydew, which is rich in minerals and antioxidants.^29,30^

#### 3.2.2 Alcohols

An important VOC class was alcohols (Figure 4), which included isoprenoids, such as hotrienol (0.07–8.78%), linalool (0.02–16.88%), and its derivatives, including *trans*-linalool oxide (0.10–3.53%) and lilac alcohol isomer (Table S1, Supporting Information). Linalool and its derivatives have been identified as components in various honey produced by A. mellifera and stingless bees.^29,31^ A study conducted by Alissandrakis et al. (2007) identified linalool and its derivatives, including *cis*- and *trans*-linalool oxides (pyranoids) and lilac aldehydes, as volatile compounds that can indicate the floral origin of citrus honey collected in Greece.^32^

Similarly, approximately ten Spanish citrus honey samples were characterized for their volatile profiles, suggesting that compounds such as linalool, linalool oxide, γ-terpineol, lilac aldehyde, and lilac alcohol isomers may serve as floral markers for citrus honey.^33^ Citrus-origin honey is characterized by the predominance of linalool derivatives, with over 80% of the volatile compounds in honey extracts identified as linalool derivatives.^34^

The M. capixaba honey (VDIMC 01) presented a higher percentage of hotrienol (8.78 ± 1.36%) when compared to other samples (Table S1, Supporting Information). Hotrienol is frequently cited as a thermal degradation product in honey.^34^ However, studies carried out by Jerkovic et al. (2010) demonstrated that this compound can occur naturally in honey due to conditions such as temperature, pH within the hive, and enzymatic action by bees.^35^

The concentration of hotrienol can increase significantly during honey maturation through the oxidative degradation of linalool or cleavage of glycosidic bonds. In immature honey, significantly lower proportions of hotrienol were compared with those in mature honey, suggesting that this compound is likely generated during honey maturation. Hotrienol may also derive from 2,6-dimethyl-3,7-octadien-2,6-diol, allylic rearrangement of 3,7-dimethyl-1,7-octadien-3,6-diol, or dehydration of (*E*) and (*Z*)-8-hydroxylinalool Therefore, the M. quadrifasciata honey, containing low levels of these volatile compounds, cannot be classified as citrus honey. This variation is influenced by the diversity of plants and food sources bees visit.^32,35^

#### 3.2.3 Chemometric Evaluation-Based GC-MS Data

Figure 5a presents the PCA score plot based on the TIC profiles from the SBH samples. In the PCA analysis, the samples are distributed in the two-dimensional plane based on variable weights to identify and recognize patterns. The M. capixaba and M. quadrifasciata honey were well-distributed across the quadrants of PC1 and PC2.

**Figure 5:**
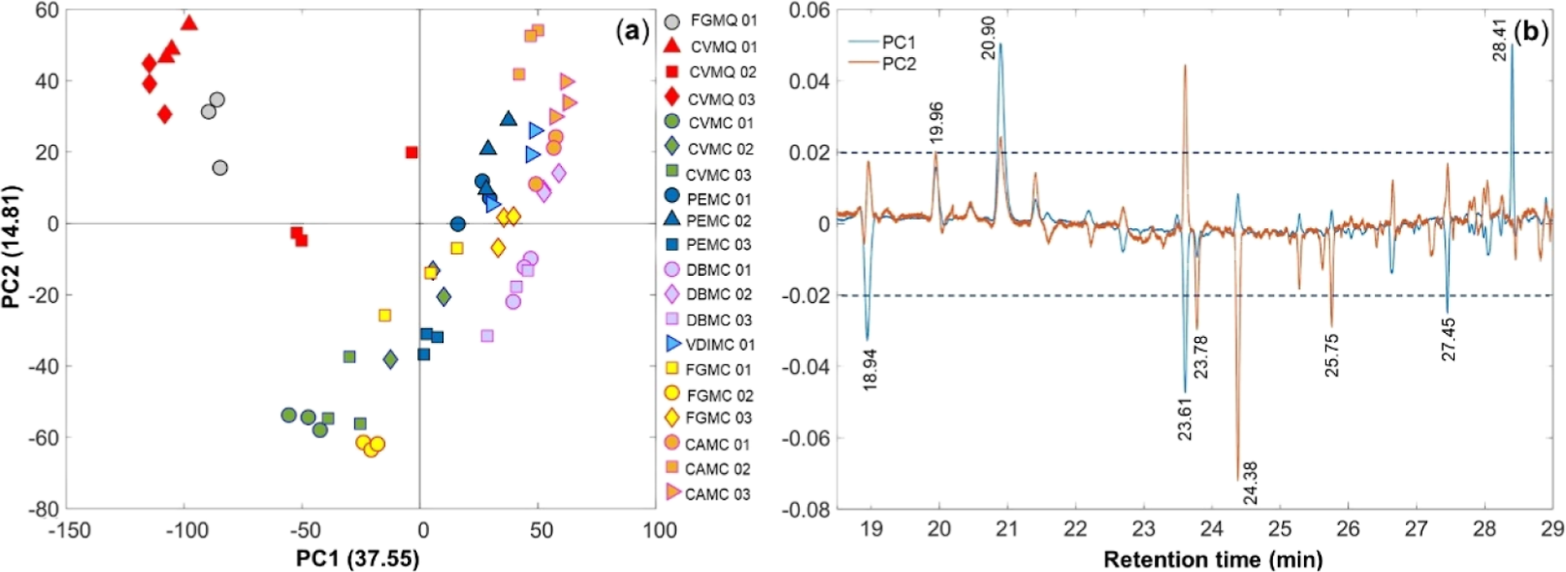
PCA plot showing PC1 *versus* PC2 from GC-MS data: (a) scores and (b) loadings plots. *t*_R_ (min): 18.94 (pentanoic acid); 19.96 (α-terpineol); 20.90 (lilac alcohol); 23.61 (ethyl dihydrocinnamate); 23.78 (benzyl alcohol); 24.38 (phenethyl alcohol); 25.75 (4-methoxybenzaldehyde); 27.45 (*n*-dodecanol); 28.41 (3,5-dimethoxybenzaldehyde).

PCA score plot shows a cumulative variance of 63.56% for three principal components (PC1, PC2, and PC3). Moreover, the PCA model showed a clear separation between M. quadrifasciata and M. capixaba SBH into two groups, with a tendency toward PC1 < 0 forM. quadrifasciata and PC1 > 0 for M. capixaba SBH group was more dispersed on PCA quadrants due to the collection location and difference in the stingless bee colonies. The weight loadings for PCA separation were pentanoic acid, lilac alcohol, ethyl dihydrocinnamate, *n*-dodecanol, and 3,5-dimethoxybenzaldehyde on PC1 and α-terpineol ethyl dihydrocinnamate, benzyl alcohol, phenethyl alcohol, and 4-methoxybenzaldehyde on PC2, as shown in Figure 5b.

These contrasts may be attributed to the availability and types of vegetation visited by stingless bees. Pattamayutanon et al. (2017) demonstrated that bees from different colonies exhibit varying contributions and preferences for specific plant types, even within the same species.^36^ Such variations in floral sources can lead to SBH samples with differences in VOC composition as well as distinct biological properties.

### 3.3 Phenolic and Flavonoid Contents

Figure 6 shows the TPC and TFC of the SBH samples. TPC varied from 6.21 ± 0.31 mg GAE 100 g^–1^ (M. quadrifasciata CVMQ 02) to 15.79 ± 0.69 mg GAE 100 g^–1^ (M. capixaba CAMC 02) even as TFC ranged from 2.37 ± 0.08 mg QE 100 g^–1^ (M. capixaba CVMQ 03) to 6.05 ± 0.39 mg QE 100 g^–1^ (M. capixaba VDIMC 01). Both TPC and TFC values were statistically different (one-way ANOVA) at a significance level of 0.05 (see Supporting Information, Table S2). The low TPC value (6.21 ± 0.21 mg GAE 100 g^–1^) and high TFC value (6.05 ± 0.40 mg QE 100 g^–1^) were those that showed the greatest difference (*p* < 0.05) within each set. M. quadrifasciata SBH presented low TPC and TFC when compared to M. capixaba. As observed, the phenolic and flavonoid values may be influenced by the bee species as well as the location and the geographical origin of nectar sources. These general features play an important key role in the variation of the polyphenol composition of honey.^37^

**Figure 6:**
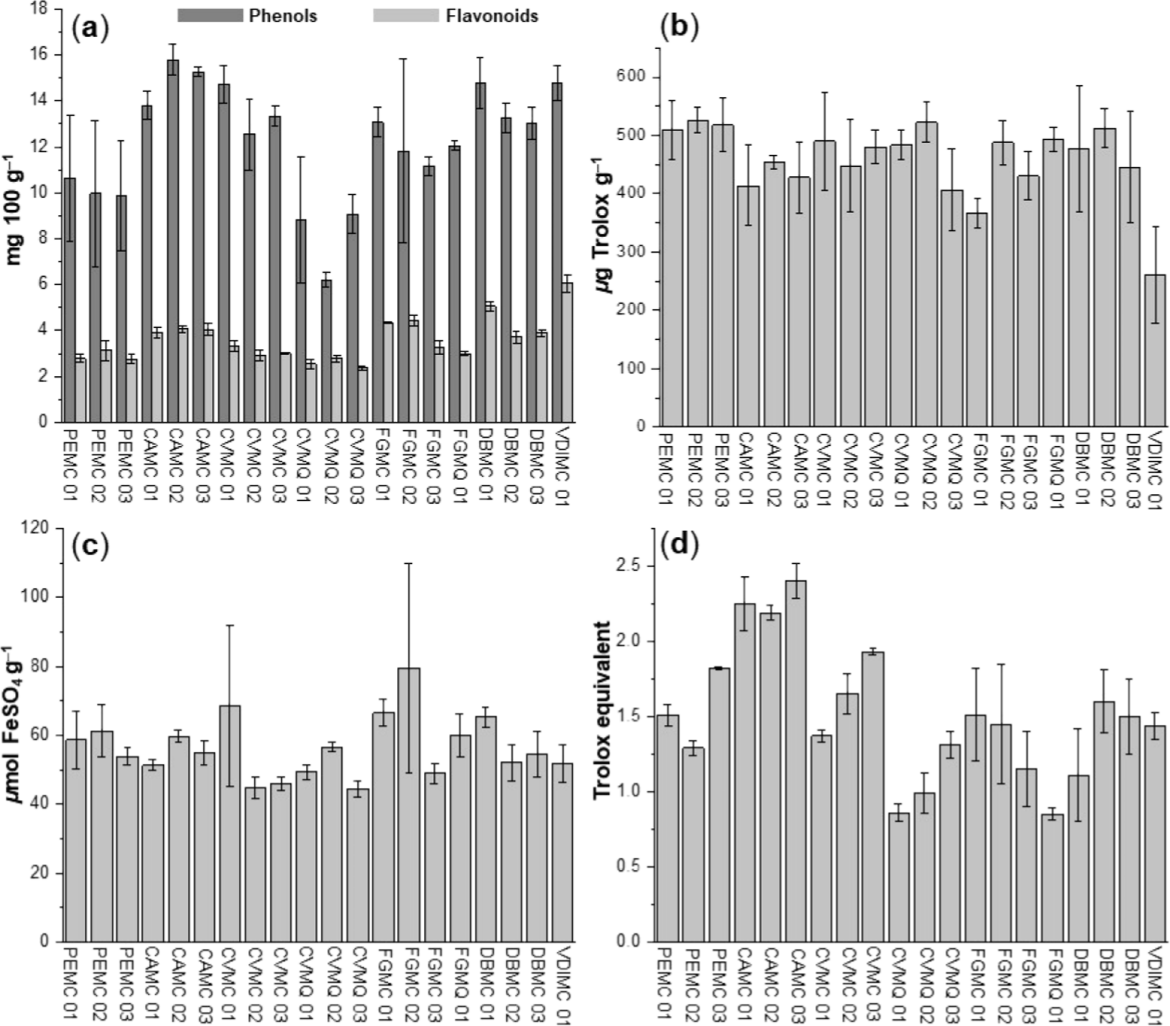
Bar chart of (a) TPC and TFC, (b) DPPH, (c) FRAP, and (d) CRAC in SBH samples. Results are presented in mean ± standard deviation.

SBH samples were grouped in PCA score plots (Figures 2a and 5a) based on stingless bee species, and the separation on PC2 may indicate a variation of chemical compounds, which includes molecules with distinct chemical groups (Figure 1) and VOCs (Figure 3). In addition, it is important to highlight that the phenol and flavonoid contents (Figure 6a) were different for SBH from different colonies (colonies 1, 2, and 3, Table 1) located in the same place. These findings can be related to the stingless bee capabilities of select plants with a specific source of secondary metabolites, especially polyphenols.

Several studies verified the importance of phenolics, especially the TPC values in SBH. Zawawi et al. (2022) determined TPC ranging from 88.3 to 132 mg GAE 100 g^–1^ for Australian honey and 22.3 to 54.2 mg GAE 100 g^–1^ for Malaysian honey.^38^ In addition, Ressutte et al. (2025) evaluated 55 samples of different stingless bee species from five states (Amazonas, Maranhão, Pará, Paná, and Rio Grande do Sul) from Brazil, and the TPC ranging from 18.80 to 135.83 mg GAE 100 g^–1^.^39^ Biluca et al. (2021) reported TPC values between 11.0 and 38.0 mg GAE 100 g^–1^ for Brazilian SBH.^40^ Our study showed that most TPC values from the Espírito Santo state exceeded 11.0 mg GAE 100 g^–1^, as shown in Figure 6a. It is important to highlight that errors with variance coefficient higher than 10% were obtained in spectrophotometric microplate reader measurements, as well as intra- and inter-reproducibility of 4.4 and 7.8%, respectively.^41^

TPC analysis measures the overall concentration of phenolic compounds, while TFC analysis specifically quantifies flavonoids, which represent a subclass of phenolic compounds. As a result, TPC values are generally higher than TFC values (Figure 6a). In addition, M. capixaba VDIMC 01 honey showed the third-highest TPC (14.80 ± 0.76 mg GAE 100 g^–1^) and the highest TFC (6.05 ± 0.39 mg QE 100 g^–1^), which suggests that the phenol profile encompasses flavonoids and other classes of phenolics, as simple phenols and hydroxyphenylpropanoid derivatives.

Flavonoids are a diverse group of phenolic compounds that contribute significantly to the biological properties of honey such as antibacterial, anti-inflammatory, and antioxidant activities. It is known that honey can have monofloral or multifloral origins and these characteristic influences both variation and concentration of phenolics, especially flavonoid types.^42^ For instance, the Heterotrigona itama honey from Malaysia presented TFC values of 25.71 mg QE 100 g^–1^ for starfruit honey, 20.67 mg QE 100 g^–1^ for gelam honey, and 10.70 mg QE 100 g^–1^ for acacia honey.^43^ Tôrres et al. (2021) analyzed TPC and TFC in M. subnitida honey, a stingless bee species native to Brazil, and reported values of 16.09 mg GAE 100 g^–1^ for TPC and 1.16 mg QE 100 g^–1^ for TFC.^44^ These findings align with those of the current study, where significantly higher TFC values were observed.

### 3.4 Antioxidant Capacity Evaluation

#### 3.4.1 DPPH Assay

DPPH radical scavenging assay was expressed as μg Trolox equivalent g^–1^ of samples where the high and low values were observed for M. capixaba PEMC02 (525.852 ± 21.85 μg TE g^–1^) andM. capixaba VDMC01 (260.59 ± 82.33 μg TE g^–1^), respectively, as shown Figure 6. All DPPH antiradical values were statistically different (*p* < 0.05), in addition, the data set presented a mean value of 457.69 ± 63.50 μg TE g^–1^.

In addition, the % DPPH radical inhibition was 12.41 and 62.73% for VDMC01 and DBMC02, respectively. It is important to highlight that variation in the contents of phenols and flavonoids was detected in SBH samples (Figure 6). In general, these compounds in honey influence antiradical responses, although other compounds may be active against DPPH radical.^45^ Xie and Schaich (2014) realized a kinetic and stoichiometric study of antioxidants and DPPH radicals and verified that the mechanism and steric factors influence the antioxidant responses.^46^

#### 3.4.2 FRAP Assay

In general, the high FRAP values in honey are associated with chemical compounds capable of reducing Fe^3+^ to Fe^2+^. The ferric reduction capacity was evaluated in SBH and the values were 44.44 ± 2.34 and 79.51 ± 30.29 μmol FeSO_4_ g^–1^ for CVMQ 03 and FGMC 02, respectively. All FRAP values were statistically different (*p* < 0.05).

The reducing ability may be related to the phenolic compounds. Biluca et al. (2020) determined the FRAP values for honey from six bee species ranging from 179.14 to 1052.74 μmol Fe^2+^ 100 g^–1^.^47^ These data show that the antioxidant activity of honey is significantly influenced by the bee species and the geographic characteristics of the production region, reflecting the complexity of its chemical composition.

FRAP values for honey of M. quadrifasciata varied from 44.44 ± 2.34 to 59.87 ± 6.30 μmol FeSO_4_ g^–1^ while M. capixaba varied from 44.69 ± 1.89 to 79.51 ± 30.29 μmol FeSO_4_ g^–1^. As observed, M. quadrifasciata showed low FRAP values, as shown in Figure 6. Similar studies have verified that M. quadrifasciata honey had low FRAP values.^48^

The high standard deviations observed in the FRAP antioxidant capacity values of the samples reflect the great intrinsic chemical variability of honey from iron-depleted bees. Furthermore, the FRAP method is sensitive to the presence of reducing compounds that react at different rates depending on their chemical structure. As a consequence, FRAP has total antioxidant capacity but does not distinguish between compounds with different reducing potentials, which may contribute to the amplification of variation in the analytical results.^49^ Another relevant aspect is the heterogeneity derived from honey as a biological matrix, composed of sugars, proteins, organic acids, and traces of minerals, which may act as interferents in the occurrence of FRAP. Small differences in the homogenization or preparation of samples before analysis may also increase this variability, as proposed by Wilczyńska (2010).^50^

#### 3.4.3 CRAC Assay

The antioxidant capacity can be determined by chronoamperometric measurements using the ability of phenolic compounds to reduce Ce^4+^ to Ce^3+^.^14^ CRAC values for each type of SBH were calculated based on the Cottrell slopes derived from the curves shown in Figure S1 (Supporting Information).

In Figure S1, most SBH samples exhibit linear trajectories with slopes lower than those of Trolox (positive control), indicating that these may potentially reduce Ce^4+^ more effectively than the reference antioxidant. The slope values obtained for the SBH samples suggest that those with TE values greater than 1.0 possess higher antioxidant capacity than water-soluble vitamin E (Trolox), the reference compound commonly used in antioxidant studies. As shown in Figure 6, CRAC values varied from 0.85 ± 0.04 to 2.40 ± 0.12 TE and the SBH with the highest TAC was the M. capixaba honey from Pedra Azul (CAMC 03, 2.40 ± 0.12 TE, Figure 6), represented by the last, cyan-colored line in Figure S1 (Supporting Information).

### 3.5 Data Correlation

Honey samples have a complex composition, containing carbohydrates, volatile organic compounds, and phenolic compounds. According to some studies, there is often a positive correlation among TPC, TFC, and TAC parameters. However, a more detailed analysis reveals exceptions to this trend, particularly in honey samples. For instance, a study by Pena Júnior et al. (2022) evaluated TAC using the DPPH method in two honey samples derived from Schinus terebinthifolius flowers.^51^ Although one sample exhibited a higher antioxidant capacity (EC_50_ = 11.30 mg mL^–1^) compared to the other (EC_50_ = 15.00 mg mL^–1^), the sample with a lower antioxidant capacity had higher TPC and TFC levels.

As observed in Figure 7, phenols (TPC) and flavonoids (TFC) had the highest Pearson correlations (*r* = 0.6758). As noted, the values of TFC were lower than TFC, indicating that other subclasses of phenolic compounds, such as simple phenolic, and hydroxyphenyl propanoid derivatives, are contained in these samples. The variation of phenols in M. quadrifasciata and M. capixaba (Figure 4) was also observed in chromatographic analysis by GC–MS with the detection of simple phenols, such as *p*-cresol and (0.02 to 0.05%) allylguaiacol (0.01 to 0.50%) derivatives (Supporting Information, Table S1).

**Figure 7:**
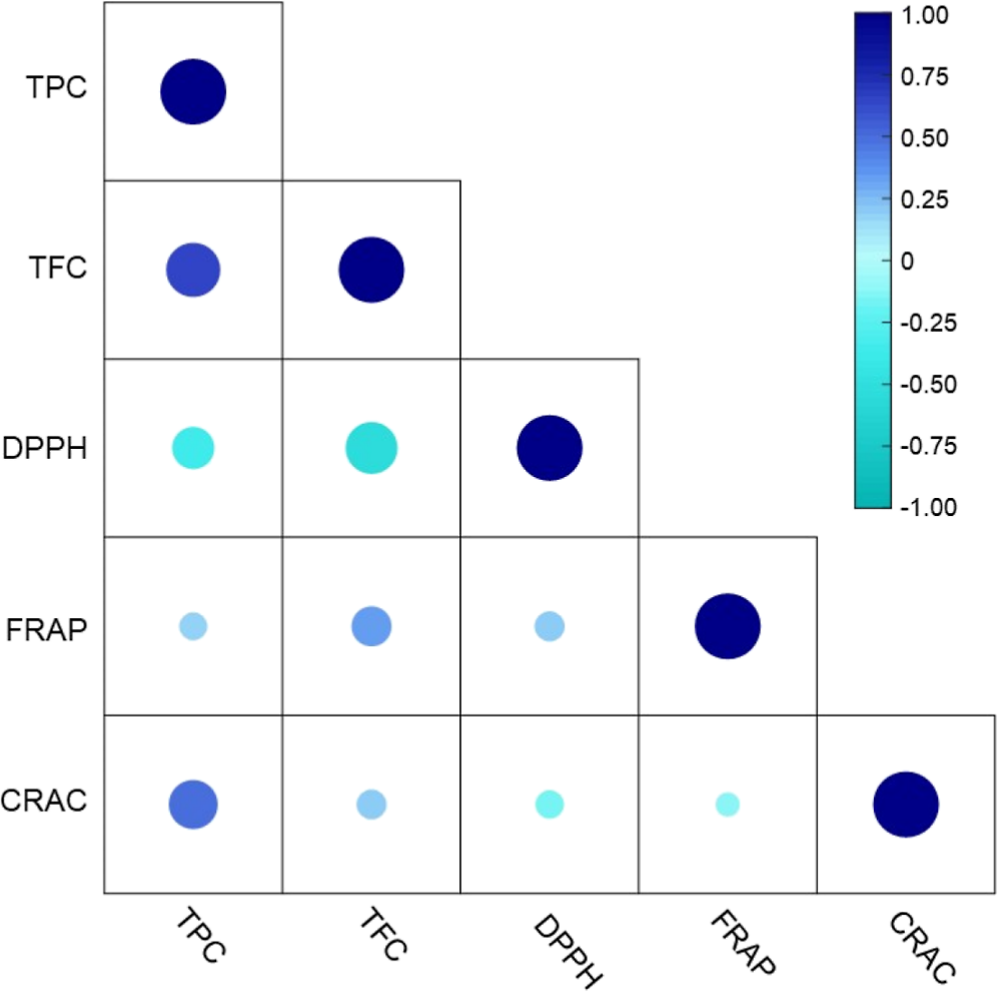
Pearson correlation coefficients (*r*) for TPC, TFC, DPPH, FRAP, and CRAC values for the stingless bee honey data set.

A study performed by Tahir et al. (2015) used statistical analyses, including Pearson correlation and PCA, which were employed to evaluate the interplay between VOCs and antioxidant parameters. The results revealed notable variations among samples from different botanical origins, suggesting that the diversity and abundance of VOCs significantly influence the antioxidant activity. For instance, alcohol and phenolic volatiles were found to have strong positive correlations with antioxidant capacity, reflecting their potential role in scavenging free radicals and contributing to overall bioactivity.^52^

It is important to highlight that TPC and TFC have different relationships with TAC parameters (Figure 7). For instance, a significant inverse Pearson correlation coefficient (*r*) was observed between DPPH and total phenolics (*r* = −0.4119) and flavonoids (*r* = −0.6203). As discussed previously, the phenolic structures have distinct molecular interactions with the DPPH radical, and their responses depend on kinetic and molecular factors, although it is expected that a high content of phenols and flavonoids have a strong Pearson correlation with DPPH. However, some studies have reported a negative and low Pearson correlation between TPC and TFC with DPPH.^53–55^ Shamsudin et al. reported strong negative Pearson correlation coefficients of −0.813 and −0.658 between TPC and DPPH, and TFC and DPPH, respectively, in SBH samples, highlighting that the inverse relationship between these variables can be associated with the contribution of synergistic and antagonist effects of chemical compounds in SBH.^55^

The FRAP assay demonstrated low Pearson correlations with TPC and TFC, *r* = 0.3699 and 0.2061, respectively, as shown in Figure 7. This weak correlation may be attributed to the low content of reducing compounds in honey as well as the significant interference of the coloration of honey on FRAP assay, as discussed in previous studies.^56^ This interference is one of the limitations of spectrophotometric assays, particularly when applied to colored or turbid samples. Although honey color can be associated with its chemical composition, including TPC and TFC, honey pigmentation can also be influenced by factors such as the presence of carotenoids, storage time, botanical origin, and contact with metallic materials during handling and postharvest processes.

Pearson correlation between the CRAC and TPC was highest when compared with the other TAC (DPPH and FRAP) with *r* = 0.5544. The high correlation observed can be attributed to the reducing capacity of the phenolic compounds present in the honey. However, the low correlation between CRAC and TFC (*r* = 0.2071) suggests that this phenolic class (flavonoids) did present significantly reducing activity. Here, the antagonistic and synergistic interactions among other components in the honey enhance its overall effectiveness. This phenomenon is particularly evident in SBH samples, where higher TPC was associated with a stronger CRAC response, which suggests that the electron transfer mechanism was predominant in the CRAC assay. It is known that simple phenolics as catechol structures have a high response in chronoamperometric measurements.^57^

The honey with the second-highest TPC value (15.26 ± 0.21 mg GAE 100 g^–1^) presented high CRAC. This effect can be attributed to the content and type of phenolics such as salicylic acid, caffeic acid, quercetin, and others, which are frequently found in SBH. These compounds were identified in Brazilian SBH in previous studies, supporting the suggested relationship between phenolic composition and TAC.^40^ Identified these compounds in Brazilian SBH, supporting the relationship between the phenolic composition and TAC. A low Pearson correlation was observed between the antioxidant capacities (Figure 7). As is known, TAC may also be influenced by two primary reaction mechanisms such as electron transfer (ET) and hydrogen atom transfer (HAT). These mechanisms can function independently or in combination, contributing to the neutralization of reactive species. The relative dominance of each mechanism often depends on the chemical structure and reactivity of the antioxidant compounds present in the system.^58^

## 4 Conclusion

This study conducted a detailed chemical characterization and a comprehensive evaluation of the antioxidant capacity of honey produced by stingless bees of theMelipona genus, collected in the state of Espírito Santo, Brazil. The results revealed a composition rich in carbohydrates, lipids, phenolic compounds, and VOCs.

M. capixaba species exhibit the highest antioxidant capacity. The use of advanced techniques such as FT-MIR, NMR, and GC-MS, combined with spectrophotometric methods FRAP and DPPH, as well as the electrochemical CRAC assay, enabled a robust evaluation of the physicochemical and bioactive properties of SBH.

The data obtained, along with the application of chemometric tools, allowed for clear differentiation of the SBH samples based on their chemical profiles and antioxidant properties. These findings highlight the nutritional and functional value of SBH. Furthermore, this study contributes to the establishment of authenticity and quality standards, essential for regulatory purposes and for promoting the sustainability and global acceptance of SBH.

## Supplementary Material



## Notes

VOCs of SBH from Espírito Santo state (Brazil) identified in TIC GC–MS, TAC, TPC, and TFC in SBH from Espírito Santo state (Brazil), and the relationship between *I* and *t* ^–1/2^ from the Cottrell equation after reaction with 0.40 g mL^–1^ of honey solutions in 0.50 mol L^–1^ H_2_SO_4_ (PDF)

## References

[1] Michener, C. D. The Meliponini In Pot-Honey, 1st ed.; Springer: New York, 2013; pp 3–17.

[2] dos
Santos A. C., Seraglio S. K. T., Gonzaga L. V., Deolindo C. T. P., Hoff R., Costa A. C. O. (2024). Brazilian stingless bee honey: A
pioneer study on the in vitro bioaccessibility of phenolic compounds. Food Chem..

[3] Ávila S., Beux M. R., Ribani R. H., Zambiazi R. C. (2018). Stingless bee honey:
Quality parameters, bioactive compounds, health-promotion properties
and modification detection strategies. Trends
Food Sci. Technol..

[4] da
Silva I. A. A., da Silva T. M. S., da Camara C. A., Queiroz N., Magnani M., de Novais J. S., Soledade L. E. B., Lima E. O., Souza A. L., Souza A. G. (2013). Phenolic
profile, antioxidant activity and palynological analysis of stingless
bee honey from Amazonas, Northern Brazil. Food
Chem..

[5] Rozman A. S., Hashim N., Maringgal N. K., Abdan A. (2022). Comprehensive Review
of Stingless Bee Products: Phytochemical Composition and Beneficial
Properties of Honey, Propolis, and Pollen. Appl.
Sci..

[6] Nayik G. A., Suhag Y. I., Majid I., Nanda V. (2018). Discrimination
of high
altitude Indian honey by chemometric approach according to their antioxidant
properties and macro minerals. J. Saudi Soc.
Agric. Sci..

[7] Ambriz-Pérez D.
L., Leyva-López N., Gutierrez-Grijalva E.
P., Heredia J. B. (2016). Phenolic
compounds: Natural alternative in inflammation treatment. A Review. Cogent Food Agric..

[8] Santos A. C. d., Biluca F. C., Braghini F., Gonzaga L. V., Costa A. C. O., Fett R. (2021). Phenolic composition and biological activities of stingless
bee honey: An overview based on its aglycone and glycoside compounds. Food Res. Int..

[9] Mokaya H. O., Bargul J. L., Irungu J. W., Lattorff H. M. G. (2020). Bioactive constituents,
in vitro radical scavenging and antibacterial activities of selected
Apis mellifera honey from Kenya. Int. J. Food
Sci. Technol..

[10] Kozłowicz K., Różyło R., Gładyszewska B., Matwijczuk A., Gładyszewsk G., Chocyk D., Samborska K., Piekut J., Smolewska M. (2020). Identifcation
of sugars and phenolic
compounds in honey powders with the use of GC–MS, FTIR spectroscopy,
and X-ray difraction. Sci. Rep..

[11] Benzie I. F. F., Strain J. J. (1996). The ferric reducing ability of plasma
(FRAP) as a measure
of “antioxidant power”: The FRAP assay. Anal. Biochem..

[12] Brand-Williams W., Cuvelier M. E. C., Berset C. (1995). Use of a free
radical method to evaluate
antioxidant activity. LWT--Food Sci. Technol..

[13] Apak R., Güçlü K., Özyürek M. S. (2008). Esin. Mechanism
of antioxidant capacity assays and the CUPRAC (cupric ion reducing
antioxidant capacity) assay. Microchim. Acta.

[14] de
Queiroz Ferreira R., Avaca L. A. (2008). Electrochemical determination of
the antioxidant capacity: The ceric reducing/antioxidant capacity
(CRAC) assay. Electroanalysis.

[15] Tappi S., Glicerina V., Ragni L., Dettori A., Romani S., Rocculi P. (2021). Physical and
structural properties of honey crystallized
by static and dynamic processes. J. Food Eng..

[16] Ganaie T. A., Masoodi F. A., Rather S. A., Wani S. M. (2021). Physicochemical,
antioxidant and FTIR-ATR spectroscopy evaluation of Kashmiri honeys
as food quality traceability and Himalayan brand. J. Food Sci. Technol..

[17] da
Silva M. C. S., da Luz J. M. R., Veloso T. G. R., Gomes W. d. S., Oliveira E. C. d. S., Anastácio L. M., Cunha Neto A., Moreli A. P., Guarçoni R. C., Kasuya M. C. M., Pereira L. L. (2022). Processing techniques and microbial
fermentation on microbial profile and chemical and sensory quality
of the coffee beverage. Eur. Food Res. Technol..

[18] Debona D. G., Lyrio M. V. V., da
Luz J. M. R., Frinhani R. Q., Araújo B. Q., Oliveira E. C. S., Agnoletti B. Z., Coura M. R., Pereira L. L., de Castro E. V. R. (2025). Comprehensive evaluation of volatile compounds and
sensory profiles of coffee throughout the roasting process. Food Chem..

[19] Suffredini H. B., Pedrosa V. A., Codognoto L., Machado S. A. S., Rocha-Filho R. C., Avaca L. A. (2004). Enhanced electrochemical
response of boron-doped diamond
electrodes brought on by a cathodic surface pre-treatment. Electrochim. Acta.

[20] Rufino, M. S. M. ; Alves, R. E. ; Brito, E. S. ; Morais, S. M. ; Sampaio, C. G. ; Pérez-Jiménez, J. ; Saura-Calixto, F. D. Determinação da atividade antioxidante total em frutas pelo método de redução do ferro (FRAP). In Comunicado Técnico; Embrapa, 2006. On-line ISSN 1679–6535.

[21] Pires, J. ; Torres, P. B. ; dos Santos, D. Y. A. C. ; Chow, F. Ensaio em Microplata do Potential Antioxidante Através do Método de Sequestro do Radical Livre DPPH Para Extratos de Algas. Instituto de Biociências, Universidade de São Paulo, 2017. ISBN 978–85–85658–71–7.

[22] Woisky R. G., Salatino A. (1998). Analysis of propolis: some parameters
and producers
for chemical quality control. J. Apic. Res..

[23] Dowd L. E. (1959). Spectrophotometric
determination of quercetin. Anal. Chem..

[24] Kasprzyk I., Depciuch J., Grabek-Lejko D., Parlinska-Wojtan M. (2018). FTIR-ATR spectroscopy
of pollen and honey as a tool for unifloral honey authentication.
The case study of rape honey. Food Control.

[25] Masek A., Chrzescijanska E., Kosmalska A., Zaborski M. (2014). Characteristics of
compounds in hops using cyclic voltammetry, UV-vis, FTIR and GC-MS
analysis. Food Chem..

[26] Krähmer A., Böttcher C., Gudi G., Stürtz M., Schulz H. (2021). Application of ATR-FTIR
spectroscopy for profiling
of non-structural carbohydrates in onion (Allium cepa L.) bulbs. Food Chem..

[27] del
Campo G., Zuriarrain J., Zuriarrain A., Berregi I. (2016). Quantitative determination of carboxylic acids, amino
acids, carbohydrates, ethanol and hydroxymethylfurfural in honey by
1H NMR. Food Chem..

[28] dos
Santos A. C., Biluca F. C., Brugnerotto F., Gonzaga L. V., Costa A. C. O., Lefft R. F. (2022). Brazilian stingless
bee honey: Physicochemical properties and aliphatic organic acids
content. Food Res. Int..

[29] Soria A. C., Sanz J., Martínez-Castro I. (2009). SPME followed by GC–MS:
a powerful technique for qualitative analysis of honey volatiles. Eur. Food Res. Technol..

[30] Shimoda M., Wu Y., Osajima Y. (1996). Aroma compounds
from aqueous solution of haze (Rhus
succedanea) honey determined by adsorptive column chromatography. J. Agric. Food Chem..

[31] Moreira R.
F. A., Trugo L. C. M., Pietroluongo M., De Maria C. A. B. (2002). Flavor Composition
of Cashew (Anacardium occidentale) and Marmeleiro (Croton Species)
Honeys. J. Agric. Food Chem..

[32] Alissandrakis E., Tarantilis P. A., Harizanis P. C., Polissiou M. (2007). Aroma investigation
of unifloral Greek citrus honey using solid-phase microextraction
coupled to gas chromatographic–mass spectrometric analysis. Food Chem..

[33] Castro-Vázquez L., Díaz-Maroto M. C., Pérez-Coello M. S. (2007). Aroma composition
and new chemical markers of Spanish citrus honeys. Food Chem..

[34] Alissandrakis E., Daferera D., Tarantilis P. A., Polissiou M., Harizanis P. C. (2003). Ultrasound-assisted extraction of
volatile compounds
from citrus flowers and citrus honey. Food Chem..

[35] Jerkovic I., Hegic G., Marijanovic Z., Bubalo D. (2010). Organic extractives
from Mentha spp. Honey and the bee-stomach: Methyl syringate, vomifoliol,
terpenediol I, hotrienol and other compounds. Molecules.

[36] Pattamayutanon P., Angeli S., Thakeow P., Abraham J., Disayathanoowat T., Chantawannakul P. (2017). Volatile organic compounds of Thai honeys produced
from several floral sources by different honey bee species. PLoS One.

[37] Biluca F. C., Braghini F., Gonzaga L. V., Costa A. C. O., Fett R. (2016). Physicochemical
profiles, minerals and bioactive compounds of stingless bee honey
(Meliponinae). J. Food Compos. Anal..

[38] Zawawi N., Zhang J., Hungerford N. L., Yates H. S. A., Webber D. C., Farrell M., Tinggi U., Bhandari B., Fletcher M. T. (2022). Unique
physicochemical properties and rare reducing sugar trehalulose mandate
new international regulation for stingless bee honey. Food Chem..

[39] Ressutte J. B., Galvan D., Luz C. F. P., Gonçalves A. M., Rebelo K. S., Sattler J. A. G., Passarinha L., Gallardo E., Anjos O., Spinosa W. A. (2025). Advanced
classification
of Brazilian stingless bee honey by genus using comprehensive analytical
techniques and chemometrics. J. Food Compos.
Anal..

[40] Carina
Biluca F., Braghini F. F. G., Campos Ferreira G., Costa R., Helena Baggio Ribeiro D., Costa dos
Santos A., Vitali L., Amadeu Micke G., Costa R., Fett R. (2020). Physicochemical parameters, bioactive
compounds, and antibacterial potential of stingless bee honey. J. Food Process..

[41] Johnson J. B., Mani J. S., Naiker M. (2022). Development
and Validation of a 96-Well
Microplate Assay for the Measurement of Total Phenolic Content in
Ginger Extracts. Food Anal. Methods.

[42] Becerril-Sánchez A. L., Quintero-Salazar B., Dublán-García, Escalona-Buendía O. H. B. (2021). Phenolic
Compounds in Honey and Their Relationship with Antioxidant Activity,
Botanical Origin, and Color. Antioxidants.

[43] Shamsudin S., Selamat J., Shomad M. A., Aziz M. F. A., Akanda M. J. H. (2022). Antioxidant
Properties and Characterization of Heterotrigona itama Honey from
Various Botanical Origins According to Their Polyphenol Compounds. J. Food Qual..

[44] Tôrres W. d. L., Vilvert J. C., Carvalho A. T., Leite R. H. d. L., Santos F. K. G. d., Aroucha E. M. M. M. (2020). Quality of Apis mellifera Honey After
Being Used in the Feeding of Jandaira Stingless Bees (Melipona subnitida). Acta Sci. Anim. Sci..

[45] Shamsudin S., Selamat J., Sanny M., Abd Razak S. B., Jambari N. N., Mian Z., Khatib A. (2019). Influence
of Origins
and Bee Species on Physicochemical, Antioxidant Properties and Botanical
Discrimination of Stingless Bee Honey. Int.
J. Food Prop..

[46] Xie J., Schaich K. M. (2014). Re-Evaluation of
the 2,2-Diphenyl-1-Picrylhydrazyl
Free Radical (DPPH) Assay for Antioxidant Activity. J. Agric. Food Chem..

[47] Carina
Biluca F., Braghini F., Campos Ferreira G., Costa dos Santos A., Helena Baggio Ribeiro D., Valdemiro
Gonzaga L., Vitali L., Amadeu Micke G., Carolina Oliveira Costa A., Fett R. (2020). Physicochemical Parameters,
Bioactive Compounds, and Antibacterial Potential of Stingless Bee
Honey. J. Food Process..

[48] dos
Santos A. C., Seraglio S. K. T., Gonzaga L. V., Deolindo C. T. P., Hoff R., Costa A. C. O. (2024). Brazilian Stingless Bee Honey: A
Pioneer Study on the In Vitro Bioaccessibility of Phenolic Compounds. Food Chem..

[49] Prior R. L., Wu X., Schaich K. (2005). Standardized
Methods for the Determination of Antioxidant
Capacity and Phenolics in Foods and Dietary Supplements. J. Agric. Food Chem..

[50] Wilczyńska A. (2010). Phenolic Content
and Antioxidant Activity of Different Types of Polish Honey –
A Short Report. J. Food Nutr. Sci..

[51] Pena
Júnior D. S., Almeida C. A., Santos M. C. F., Fonseca P. H. V., Menezes E. V., de Melo Junior A. F., Brandão M. M., de Oliveira D. A., Souza L. F. d., Silva J. C., Royo V. d. A. (2022). Antioxidant
Activities of Some Monofloral Honey Types Produced Across Minas Gerais
(Brazil). PLoS One.

[52] Tahir H. E., Xiaobo Z., Zhihua L., Yaodi Z. (2015). Comprehensive Evaluation
of Antioxidant Properties and Volatile Compounds of Sudanese Honeys. Food Biochem..

[53] Sant’ana L. D., Ferreira M. C. A., Lorezon R. L. L., Berbara R. N. (2014). Correlation of Total
Phenolic and Flavonoid Contents of Brazilian Honey with Color and
Antioxidant Capacity. Int. J. Food Prop..

[54] Pontis J. A., Costa L. A. M. A. d., Silva S. J. R., Flach A. (2014). Color, phenolic and
flavonoid content, and antioxidant activity of honey from Roraima,
Brazil. Food Sci. Technol..

[55] Shamsudin S., Selamat J., Shomad M. A., Aziz M. F. A., Akanda M. J. H. (2022). Antioxidant
Properties and Characterization of Heterotrigona itama Honey from
Various Botanical Origins According to Their Polyphenol Compounds. J. Food Qual..

[56] Haque M. A., Morozova K., Ferrentino G., Scampicchio M. (2021). Electrochemical
Methods to Evaluate the Antioxidant Activity and Capacity of Foods:
A Review. Electroanalysis.

[57] Aravindan N., Preethi S., Sangaranarayanan M. V. (2017). Non-Enzymatic Selective Determination
of Catechol Using Copper Microparticles Modified Polypyrrole Coated
Glassy Carbon Electrodes. J. Electrochem. Soc..

[58] Apak R., Özyürek M., Güçlü K., Çapanoğlu E. (2016). Antioxidant Activity/Capacity Measurement.
1. Classification, Physicochemical Principles, Mechanisms, and Electron
Transfer (ET)-Based Assays. J. Agric. Food Chem..

