# Supplementary material for: Influence of Chemical Profile on the Antioxidant Capacity of Brazilian Stingless Bee Honey
Source: ACS Omega. 2025 May 12;10(20):20550–61. doi: 10.1021/acsomega.5c01134 (PMC12120604; doi:10.1021/acsomega.5c01134)
Supplement: Supplementary file 1 [file ao5c01134_si_001.pdf]

Influence of Chemical Profile on Antioxidant Capacity of Brazilian Stingless Bee Honey

Lucas R. de O. Dias[1], Bruna M. Damm[1], Bruno F. Paqueli[1], Bruno Q. Araújo[1], Gislane C. Oliveira[2], Diolina M. Silva[2], Eustaquio V. R. de Castro[1], Rafael de Q. Ferreira[1,*], Álvaro C. Neto[1]

[1]Chemistry Department, Federal University of Espírito Santo, 29075-910, Vitória, Espírito Santo, Brazil

[2]Post-graduate Program of Vegetal Biology, Federal University of Espírito Santo, 29075-910, Vitória, Espírito Santo, Brazil

*Corresponding author: rafael.q.ferreira@ufes.br; Department of Chemistry, Federal University of Espírito Santo, 29075-910 Vitória – ES, Brazil; Tel.: +55 27 31455417; fax: +55 27 40092826.

Supporting information

The supplementary material presents Table S1, which contains the volatile organic compounds of SBH from Espírito Santo state (Brazil) identified in TIC GC-MS, Table S2, which shows the total antioxidant capacity, TPC, and TFC in SBH from Espírito Santo state (Brazil), and Figure S1, which presents the relationship between I and $t^{-1/2}$ from the Cottrell equation after reaction with 0.40 g mL^{-1} of honey solutions in 0.50 mol L^{-1} H_2SO_4.

Table S1. Volatile organic compounds (VOCs, mean ± standard deviation) of SBH from Espírito Santo state (Brazil) identified in TIC GC–MS.

No	VOCs	RI *	t_R (min)	PEMC 01	PE MC 02	PE MC 03	CAM C 01	CAM C 02	CAM C 03	CVM C 01	CV MC 02	CV MC 03	CVM Q 01	CVM Q 02	CV MQ 03	FG MC 01	FG MC 02	FG MC 03	FGMQ 01	DBMC 01	DB MC 02	DB MC 03	VDI MC 01
1	propanol	1012	3.186	0.99 ± 0.01	0.88 ± 0.33	0.31 ± 0.06	0.74 ± 0.51	0.42 ± 0.11	0.33 ± 0.01	1.06 ± 0.65	0.37 ± 0.31	0.78 ± 0.71		0.99 ± 0.14			0.38 ± 0.06	1.00 ± 0.23		0.32 ± 0.10	0.24 ± 0.07	0.37 ± 0.20	1.20 ± 0.35
2	NI	1073	3.502												0.09 ± 0.01				0.33 ± 0.13				
3	NI	1096	3.843															0.07 ± 0.05					
4	isobutyl alcohol	1115	4.124	0.85 ± 0.00	0.67 ± 0.34	0.11 ± 0.08	0.19 ± 0.15	0.26 ± 0.09	0.37 ± 0.06	0.37 ± 0.34		0.14 ± 0.06	0.99 ± 0.21	0.85 ± 0.15	1.55 ± 0.65	0.38 ± 0.32	0.53 ± 0.38	0.18 ± 0.07	0.99 ± 0.51	0.04 ± 0.06	0.19 ± 0.06		
5	heptane-2,3-dione	1140	4.504	1.03 ± 0.01	0.71 ± 0.27	0.23 ± 0.04	0.26 ± 0.07		0.11 ± 0.02	0.72 ± 0.44	0.36 ± 0.13	0.16 ± 0.07	0.26 ± 0.03	1.03 ± 0.10	0.61 ± 0.18	2.21 ± 1.96	1.03 ± 0.22	0.16 ± 0.01	2.04 ± 0.83	0.18 ± 0.06	0.19 ± 0.04	0.10 ± 0.05	0.24 ± 0.05
6	limonene	1169	5.151					0.06 ± 0.04											0.08 ± 0.02				
7	NI	1211	6.093																		0.15 ± 0.05		0.14 ± 0.01
8	β-cis-ocimene	1215	6.195					0.07 ± 0.02				0.13 ± 0.03											
9	NI	1222	6.342													0.12 ± 0.05		0.06 ± 0.04					
10	isoamylalcohol	1240	6.756	2.67 ± 0.01	2.60 ± 1.51	0.95 ± 0.20			0.85 ± 0.01	1.78 ± 1.68	2.16 ± 1.14	1.82 ± 1.62				2.20 ± 1.98	1.23 ± 0.97			0.02 ± 0.02	0.49 ± 0.13	0.33 ± 0.14	0.17 ± 0.10
11	NI	1244	6.859				0.57 ± 0.21	1.04 ± 0.43						0.11 ± 0.02	2.11 ± 1.49			0.48 ± 0.03	1.63 ± 1.14				
12	isobutenylcarbinol	1273	7.677					0.06 ± 0.04															
13	acetoin	1297	8.354	32.04 ± 0.03	11.3 ± 0.78	4.33 ± 0.50	2.17 ± 0.32	1.26 ± 0.02	3.00 ± 0.03	2.50 ± 1.79	4.30 ± 1.05	1.90 ± 0.61	1.34 ± 0.59			20.0 ± 11.44	6.97 ± 0.45	0.66 ± 0.05	10.45 ± 1.02	2.91 ± 0.22	3.87 ± 0.06	0.53 ± 0.47	4.30 ± 0.13

14	NI	1301	8.462										3.90 ± 0.21	0.70 ± 0.14	9.96 ± 0.56								
15	methyl acetate	1306	8.589	0.19 ± 0.03	0.30 ± 0.05	0.73 ± 0.09		0.34 ± 0.06	0.30 ± 0.02		0.43 ± 0.14	0.55 ± 0.12		0.14 ± 0.01		0.32 ± 0.06	0.17 ± 0.01			0.41 ± 0.11	0.46 ± 0.02	0.78 ± 0.16	0.08 ± 0.04
16	acetol	1309	8.64				0.35 ± 0.10								0.08 ± 0.02			0.11 ± 0.03	0.09 ± 0.03				
17	NI	1342	9.618							0.16 ± 0.26							1.78 ± 2.48					0.06 ± 0.07	0.45 ± 0.07
18	methoxyacetic acid 2-pentyl ester	1344	9.663			0.07 ± 0.03	0.10 ± 0.01			0.20 ± 0.08	0.81 ± 0.34	0.11 ± 0.06								0.29 ± 0.09		0.14 ± 0.18	0.67 ± 0.11
19	NI	1346	9.734				0.24 ± 0.22		0.07 ± 0.01			0.08 ± 0.03					1.67 ± 2.17				0.10 ± 0.01		
20	2-hydroxy-propanoic acid ethyl ester	1349	9.816	2.81 ± 0.01	7.53 ± 2.52	0.56 ± 0.05			0.21 ± 0.02				10.52 ± 1.95		13.68 ± 2.23			7.16 ± 0.56	12.45 ± 4.51	5.07 ± 0.33	0.57 ± 0.03		
21	2-methyl-hexan-2-ol	1355	10.003	0.55 ± 0.00					0.02 ± 0.04	0.14 ± 0.19						5.11 ± 3.86	0.37 ± 0.18		0.69 ± 0.75				0.17 ± 0.06
22	NI	1361	10.157						0.02 ± 0.02								0.03 ± 0.03						
23	NI	1375	10.569						0.03 ± 0.03								0.02 ± 0.02			0.05 ± 0.04			
24	NI	1386	10.897																				0.14 ± 0.05
25	(Z)-3-hexenol	1397	11.201					0.02 ± 0.03	0.03 ± 0.11											0.05 ± 0.01			
26	NI	1419	11.859														0.03 ± 0.03						
27	NI	1429	12.137									0.07 ± 0.00					0.02 ± 0.03						
28	NI	1443	12.552	2.04 ± 0.01	0.60 ± 0.21	0.14 ± 0.03	0.27 ± 0.07	0.05 ± 0.00	0.36 ± 0.00	0.21 ± 0.30	0.09 ± 0.09	0.04 ± 0.07	0.02 ± 0.02	0.11 ± 0.02	0.08 ± 0.03	1.51 ± 1.18				0.27 ± 0.11	0.27 ± 0.03	0.04 ± 0.03	0.89 ± 0.19

29	acetic acid	1448	12.691	45.86 ± 0.00	64.78 ± 8.68	85.19 ± 1.99	75.54 ± 4.17	58.19 ± 6.08	60.91 ± 0.03	85.71 ± 5.14	84.73 ± 3.78	88.63 ± 4.76	75.86 ± 3.74	77.24 ± 2.65	65.71 ± 4.51	47.65 ± 34.50	77.52 ± 0.81	82.7 ± 1.00	63.60 ± 10.52	73.35 ± 0.92	75.24 ± 2.65	83.04 ± 2.23	63.54 ± 3.45
30	2-hydroxy-4-methyl-pentanoic acid methyl ester	1465	13.173	0.84 ± 0.01	0.20 ± 0.07	0.10 ± 0.02	0.04 ± 0.04		0.03 ± 0.04	0.12 ± 0.03	0.10 ± 0.06					1.23 ± 1.17	0.32 ± 0.11		0.25 ± 0.08	0.08 ± 0.02	0.06 ± 0.01		0.13 ± 0.03
31	NI	1473	13.424					0.94 ± 1.58	0.02 ± 0.01														
32	linalool oxide isomer	1481	13.663	0.66 ± 0.01	1.41 ± 0.51	0.27 ± 0.06	3.00 ± 0.39	1.80 ± 1.58	4.23 ± 0.01		0.24 ± 0.12	0.12 ± 0.01	0.28 ± 0.15	0.59 ± 0.03	0.10 ± 0.05	2.17 ± 1.71	0.24 ± 0.03	0.62 ± 0.06	0.18 ± 0.08	3.53 ± 0.77	2.76 ± 0.28	1.54 ± 0.73	3.53 ± 0.64
33	benzaldehyde	1498	14.134	0.19 ± 0.00	1.10 ± 0.48	0.16 ± 0.06	1.12 ± 0.36	1.04 ± 0.32	0.81 ± 0.03	0.35 ± 0.28	0.81 ± 0.29	0.45 ± 0.30	0.04 ± 0.01	0.12 ± 0.04	0.05 ± 0.03	1.20 ± 1.18	0.41 ± 0.05	0.36 ± 0.02	0.10 ± 0.07	0.29 ± 0.05	0.58 ± 0.18	0.53 ± 0.17	0.96 ± 0.24
34	NI	1523	14.864					0.02 ± 0.01									0.03 ± 0.03				0.06 ± 0.01		
35	NI	1531	15.1					0.08 ± 0.02	0.04 ± 0.04										0.10 ± 0.05				0.09 ± 0.02
36	propanoic acid	1539	15.319	1.67 ± 0.01	1.90 ± 0.19	1.32 ± 0.14	0.88 ± 0.08	0.51 ± 0.03	0.98 ± 0.02	1.53 ± 0.28	1.55 ± 0.66	2.34 ± 0.67	0.71 ± 0.05	1.50 ± 0.25	0.59 ± 0.05	1.73 ± 0.82	1.14 ± 0.01	2.40 ± 0.05	1.09 ± 0.12	0.97 ± 0.16	1.36 ± 0.06	1.05 ± 0.07	2.76 ± 0.25
37	linalool	1555	15.753	0.02 ± 0.02		0.06 ± 0.07	5.63 ± 1.87	16.88 ± 5.10	12.02 ± 0.02	0.05 ± 0.04				4.10 ± 0.11		4.13 ± 0.11	0.51 ± 0.03	0.15 ± 0.01		5.08 ± 1.34	4.47 ± 1.68	3.52 ± 1.12	3.72 ± 0.95
38	2-methylpropanoic acid	1570	16.153	0.15 ± 0.01	0.12 ± 0.06	0.08 ± 0.02	0.07 ± 0.03	0.06 ± 0.00	0.08 ± 0.01	0.14 ± 0.04	0.12 ± 0.04	0.09 ± 0.05	0.71 ± 0.11	0.13 ± 0.01	0.71 ± 0.08	0.27 ± 0.21	0.13 ± 0.03	0.06 ± 0.01	0.25 ± 0.09	0.09 ± 0.00	0.08 ± 0.01	0.05 ± 0.02	0.09 ± 0.01
39	NI	1577	16.339	0.06 ± 0.01	0.07 ± 0.01	0.05 ± 0.02	0.05 ± 0.04	0.07 ± 0.02	0.06 ± 0.01	0.11 ± 0.05	0.07 ± 0.02	0.07 ± 0.01			0.05 ± 0.01	0.07 ± 0.01	0.07 ± 0.03	0.05 ± 0.02	0.05 ± 0.01	0.08 ± 0.00	0.05 ± 0.01	0.08 ± 0.01	0.10 ± 0.02
40	NI	1590	16.687	0.08 ± 0.02	0.09 ± 0.03	0.09 ± 0.01	0.04 ± 0.03	0.04 ± 0.01	0.05 ± 0.03	0.13 ± 0.05	0.13 ± 0.02	0.10 ± 0.02	0.04 ± 0.00	0.05 ± 0.03	0.09 ± 0.02	0.09 ± 0.03	0.10 ± 0.03	0.07 ± 0.02	0.07 ± 0.01	0.09 ± 0.01	0.07 ± 0.02	0.08 ± 0.01	0.10 ± 0.03
41	3,5,5-trimethyl-cyclohex-2-enone	1606	17.128					0.03 ± 0.02									0.04 ± 0.02						
42	NI	1611	17.239							0.04 ± 0.02	0.05 ± 0.03	0.11 ± 0.01											
43	hotrienol	1615	17.368	2.94 ± 0.01	1.02 ± 0.40	1.16 ± 0.34	1.61 ± 0.36			0.18 ± 0.12	0.32 ± 0.30	0.08 ± 0.05	0.07 ± 0.04		0.72 ± 0.60	1.81 ± 0.61	2.16 ± 0.28	0.50 ± 0.38	0.87 ± 0.44		1.62 ± 0.29		8.78 ± 1.36

44	benzeneacetaldehyde	1618	17.428					4.37 ± 1.61	6.39 ± 0.03											4.15 ± 2.02		2.07 ± 0.48	
45	butanoic acid	1633	17.826	0.06 ± 0.00		0.08 ± 0.04	0.23 ± 0.04	0.05 ± 0.07	0.12 ± 0.04	0.13 ± 0.02	0.12 ± 0.04	0.15 ± 0.07	0.17 ± 0.04			0.13 ± 0.10	0.09 ± 0.06	0.09 ± 0.02		0.06 ± 0.05	0.14 ± 0.05	0.08 ± 0.06	
46	ethylbenzoate	1648	18.165				0.06 ± 0.04						0.04 ± 0.01						0.06 ± 0.02				
47	pentanoic acid	1690	18.942	0.39 ± 0.02	0.29 ± 0.09	0.18 ± 0.01	0.12 ± 0.04	0.04 ± 0.04	0.15 ± 0.00	0.26 ± 0.11	0.35 ± 0.17	0.15 ± 0.09	1.00 ± 0.14	0.31 ± 0.02	0.55 ± 0.11	0.85 ± 0.61	0.24 ± 0.07	0.09 ± 0.03	1.37 ± 0.46	0.14 ± 0.03	0.13 ± 0.02	0.05 ± 0.04	0.16 ± 0.06
48	NI	1698	19.095					0.03 ± 0.03												0.02 ± 0.01			
49	α-terpineol	1751	20.034				0.43 ± 0.02	1.39 ± 0.21	0.27 ± 0.02				0.04 ± 0.01	0.34 ± 0.02	0.02 ± 0.02	0.03 ± 0.02	0.12 ± 0.02	0.05 ± 0.01	0.04 ± 0.01	0.26 ± 0.23	0.44 ± 0.10	0.18 ± 0.30	0.21 ± 0.01
50	lilac alcohol isomer	1781	20.461				0.20 ± 0.03	0.22 ± 0.06	0.34 ± 0.01				0.05 ± 0.01			0.06 ± 0.04				0.20 ± 0.03	0.16 ± 0.04	0.13 ± 0.05	0.25 ± 0.05
51	NI	1796	20.672						0.17 ± 0.48														
52	epoxylinalol isomer	1813	20.906	1.69 ± 0.01	2.32 ± 0.77	0.96 ± 0.18	2.61 ± 0.50	1.41 ± 0.69	3.65 ± 0.01	0.16 ± 0.04	0.82 ± 0.33	0.24 ± 0.13	0.43 ± 0.10	2.22 ± 0.14	0.15 ± 0.03	0.38 ± 0.19	0.18 ± 0.06	0.98 ± 0.04	0.41 ± 0.14	2.75 ± 0.36	2.42 ± 0.14	1.51 ± 0.40	2.85 ± 0.35
53	ethyl phenylacetate	1853	21.434	0.07 ± 0.03	0.06 ± 0.03	0.04 ± 0.04	0.05 ± 0.01	1.20 ± 0.07	0.23 ± 0.01	0.11 ± 0.05			0.02 ± 0.01			0.10 ± 0.07	0.02 ± 0.01	0.06 ± 0.02	0.06 ± 0.02		0.09 ± 0.02		
54	epoxylinalol isomer	1867	21.606	0.15 ± 0.01	0.13 ± 0.07	0.10 ± 0.01	0.12 ± 0.03	0.08 ± 0.01	0.16 ± 0.01		0.16 ± 0.08	0.09 ± 0.04	0.02 ± 0.01	0.10 ± 0.03		1.14 ± 0.86	0.04 ± 0.02	0.09 ± 0.00	0.06 ± 0.01	0.12 ± 0.03	0.10 ± 0.02	0.11 ± 0.03	0.20 ± 0.03
55	β-citronellol	1880	21.761					0.08 ± 0.02															
56	lilac alcohol isomer	1917	22.199			0.03 ± 0.02	0.18 ± 0.07	0.18 ± 0.03	0.19 ± 0.01	0.07 ± 0.05	0.06 ± 0.02	0.04 ± 0.00	0.02 ± 0.00	0.07 ± 0.05		0.10 ± 0.06	0.08 ± 0.03		0.04 ± 0.01	0.20 ± 0.01	0.25 ± 0.00		0.18 ± 0.00
57	NI	1933	22.387					0.01 ± 0.01														0.10 ± 0.13	
58	NI	1940	22.48					0.06 ± 0.01	0.02 ± 0.00				0.07 ± 0.01	0.06 ± 0.02	0.13 ± 0.03		0.11 ± 0.16		0.34 ± 0.13			0.02 ± 0.02	
59	NI	1954	22.622					0.01 ± 0.00	0.01 ± 0.00				0.04 ± 0.01										

60	NI	1988	22.984													0.16 ± 0.12		0.06 ± 0.02					0.07 ± 0.01
61	lilac alcohol isomer	2002	23.128				0.06 ± 0.01	0.11 ± 0.00	0.10 ± 0.02				0.92 ± 0.14	0.06 ± 0.01	0.04 ± 0.01	0.14 ± 0.12	0.02 ± 0.02		0.06 ± 0.03	0.07 ± 0.02	0.05 ± 0.01	0.06 ± 0.00	0.10 ± 0.03
62	NI	2017	23.286				0.03 ± 0.01		0.01 ± 0.01		0.03 ± 0.01	0.03 ± 0.01				0.12 ± 0.17	0.03 ± 0.02			0.03 ± 0.01	0.03 ± 0.01	0.02 ± 0.02	0.05 ± 0.01
63	trans-geraniol	1847	23.496				0.07 ± 0.01	0.25 ± 0.04	0.10 ± 0.01					0.05 ± 0.01		0.05 ± 0.04	0.02 ± 0.00			0.07 ± 0.01	0.11 ± 0.01	0.18 ± 0.02	
64	ethyl 3-phenylpropionate	1857	23.598					0.03 ± 0.01															
65	benzyl alcohol	1876	23.78	0.15 ± 0.01	0.17 ± 0.05	0.14 ± 0.04	0.07 ± 0.03	0.04 ± 0.02	0.10 ± 0.02	0.30 ± 0.08		0.18 ± 0.03	0.02 ± 0.00	0.38 ± 0.08	0.04 ± 0.01		0.04 ± 0.00	0.05 ± 0.01	0.06 ± 0.03	0.08 ± 0.01		0.07 ± 0.01	
66	NI	1880	23.825								0.17 ± 0.05		0.01 ± 0.00			0.04 ± 0.01	0.04 ± 0.00		0.04 ± 0.01		0.09 ± 0.01		0.06 ± 0.02
67	NI	1889	23.904										0.14 ± 0.02	0.11 ± 0.02	0.03 ± 0.02		0.01 ± 0.01						0.04 ± 0.00
68	NI	1915	24.162							0.03 ± 0.02						0.01 ± 0.01							
69	NI	1923	24.236													0.07 ± 0.04			0.04 ± 0.02				
70	NI	1929	24.299							0.14 ± 0.21						0.05 ± 0.05							
71	phenethyl alcohol	1939	24.391	0.36 ± 0.01	0.28 ± 0.11	0.43 ± 0.04	0.42 ± 0.04	0.06 ± 0.03	0.34 ± 0.01	0.19 ± 0.16	0.45 ± 0.12	0.23 ± 0.07	0.08 ± 0.09	0.75 ± 0.05	0.17 ± 0.03			0.28 ± 0.01	0.17 ± 0.01	1.73 ± 0.07	0.77 ± 0.05	1.26 ± 0.13	0.23 ± 0.07
72	2-phenyl-2-butenal	1946	24.464					0.22 ± 0.01															
73	NI	1966	24.647						0.02 ± 17.30							0.01 ± 0.01	0.60 ± 0.09						
74	NI	1974	24.721													0.04 ± 0.04							

75	NI	1989	24.85													0.19 ± 0.19							
76	NI	2002	24.973													0.02 ± 0.01							
77	NI	2011	25.055													0.03 ± 0.02							
78	NI	2021	25.145							0.05 ± 0.04						0.08 ± 0.07							
79	NI	2029	25.209							0.10 ± 0.03	0.08 ± 0.02					0.11 ± 0.10		0.09 ± 0.04					
80	2,6-dimethyl-octa-3,7-diene-2,6-diol	2038	25.292	0.18 ± 0.01	0.15 ± 0.02	0.32 ± 0.02	0.10 ± 0.03	0.08 ± 0.01	0.10 ± 0.01			0.06 ± 0.01	0.02 ± 0.00	0.06 ± 0.02	0.03 ± 0.01	0.11 ± 0.11		0.03 ± 0.01	0.07 ± 0.02	0.08 ± 0.02	0.08 ± 0.02	0.10 ± 0.04	1.04 ± 0.35
81	NI	2095	25.358								0.06 ± 0.02					0.03 ± 0.02	0.15 ± 0.04		0.04 ± 0.01				
82	NI	2127	25.631	0.06 ± 0.01			0.04 ± 0.02		0.04 ± 0.01	0.08 ± 0.04	0.06 ± 0.01	0.04 ± 0.02	0.02 ± 0.00			0.16 ± 0.10		0.13 ± 0.00	0.03 ± 0.00	0.06 ± 0.01	0.05 ± 0.01	0.04 ± 0.00	0.05 ± 0.01
83	NI	2143	25.77	0.38 ± 0.01	0.23 ± 0.02	0.49 ± 0.07	0.41 ± 0.02	0.03 ± 0.01	0.05 ± 0.02	0.05 ± 0.02	0.03 ± 0.01	0.05 ± 0.01	0.03 ± 0.00	0.06 ± 0.02		0.47 ± 0.29	0.09 ± 0.01		0.05 ± 0.01	0.05 ± 0.01	0.04 ± 0.01	0.03 ± 0.01	0.05 ± 0.02
84	NI	2158	25.894				0.04 ± 0.01												0.01 ± 0.00				
85	trans-nerolidol	2065	25.96				0.05 ± 0.00	0.04 ± 0.01	0.04 ± 0.01	0.03 ± 0.02			0.03 ± 0.01	0.02 ± 0.00	0.03 ± 0.01		0.26 ± 0.01		0.02 ± 0.00	0.03 ± 0.00	0.07 ± 0.01	0.04 ± 0.01	
86	NI	2035	26.043										0.03 ± 0.01		0.04 ± 0.00			0.01 ± 0.00	0.02 ± 0.01				
87	octanoic acid	2059	26.249	0.03 ± 0.01			0.02 ± 0.01		0.06 ± 0.01				0.07 ± 0.00	0.08 ± 0.01	0.06 ± 0.01	0.04 ± 0.03		0.02 ± 0.01			0.02 ± 0.00		
88	NI	2071	26.341													0.02 ± 0.02							
89	p-cresol	2081	26.428	0.02 ± 0.01			0.05 ± 0.00			0.04 ± 0.02	0.02 ± 0.01	0.03 ± 0.01				0.03 ± 0.02					0.03 ± 0.01		

90	NI	2091	26.505				0.04 ± 0.01											0.01 ± 0.00					
91	NI	2107	26.632	0.03 ± 0.01			0.03 ± 0.01	0.02 ± 0.01	0.05 ± 0.01	0.03 ± 0.01	0.02 ± 0.01		0.22 ± 0.03	0.20 ± 0.02	0.22 ± 0.02	0.06 ± 0.05	0.02 ± 0.01		0.10 ± 0.05	0.03 ± 0.00	0.04 ± 0.01	0.04 ± 0.01	0.05 ± 0.00
92	NI	2115	26.695	0.02 ± 0.01	0.03 ± 0.00	0.04 ± 0.01	0.08 ± 0.02			0.03 ± 0.02	0.03 ± 0.02	0.02 ± 0.00		0.02 ± 0.01			0.03 ± 0.00	0.02 ± 0.01		0.02 ± 0.00			
93	NI	2122	26.752								0.04 ± 0.01					0.05 ± 0.06	0.02 ± 0.01				0.03 ± 0.01		
94	rosifoliol	2158	27.042										0.03 ± 0.00										
95	allylguaiacol	2179	27.206				0.05 ± 0.01	0.01 ± 0.01	0.04 ± 0.00	0.04 ± 0.01	0.09 ± 0.02	0.05 ± 0.00				0.50 ± 0.32	0.02 ± 0.00	0.02 ± 0.01		0.05 ± 0.02			
96	NI	2181	27.225					0.05 ± 0.03															
97	p-ethylphenol	2198	27.36	0.03 ± 0.01	0.04 ± 0.02	0.06 ± 0.02	0.06 ± 0.02	0.02 ± 0.01	0.05 ± 0.01				0.04 ± 0.01		0.04 ± 0.01	0.04 ± 0.02	0.05 ± 0.05	0.02 ± 0.00	0.03 ± 0.01				
98	dodecanol	2209	27.445	0.05 ± 0.00	0.10 ± 0.03	0.09 ± 0.01	0.83 ± 0.12	0.09 ± 0.03	0.07 ± 0.01	0.11 ± 0.06	0.04 ± 0.03	0.05 ± 0.02	0.46 ± 0.04	0.09 ± 0.04	0.47 ± 0.00	0.03 ± 0.10	0.02 ± 0.01		0.17 ± 0.05	0.05 ± 0.01	0.08 ± 0.01	0.07 ± 0.01	0.08 ± 0.04
99	NI	2221	27.534										0.01 ± 0.00		0.01 ± 0.00				0.01 ± 0.00				
100	NI	2230	27.608					0.01 ± 0.00															
101	NI	2238	27.671					0.01 ± 0.00					0.03 ± 0.01	0.04 ± 0.01	0.03 ± 0.02		0.05 ± 0.01		0.07 ± 0.07		0.02 ± 0.00		
102	agarospirol	2252	27.779										0.05 ± 0.01		0.07 ± 0.01								
103	NI	2272	27.928	0.02 ± 0.02								0.04 ± 0.01	0.05 ± 0.01	0.06 ± 0.01	0.07 ± 0.01					0.02 ± 0.01			0.05 ± 0.02
104	ethyl hexadecanoate	2286	28.039	0.03 ± 0.00	0.07 ± 0.02	0.02 ± 0.01	0.03 ± 0.01	0.05 ± 0.04					0.07 ± 0.01				0.02 ± 0.00	0.03 ± 0.01	0.16 ± 0.22				

105	NI	2293	28.09	0.02 ± 0.00	0.11 ± 0.02	0.06 ± 0.02	0.05 ± 0.02	0.02 ± 0.01	0.07 ± 0.00	0.05 ± 0.03	0.03 ± 0.01	0.04 ± 0.02		0.05 ± 0.02			0.02 ± 0.01	0.01 ± 0.00		0.04 ± 0.02	0.04 ± 0.01	0.87 ± 0.07	0.05 ± 0.03
106	NI	2304	28.172				0.08 ± 0.02											0.01 ± 0.00					
107	trimethoxy benzene	2319	28.283				0.04 ± 0.02	0.10 ± 0.01	0.16 ± 0.01	0.04 ± 0.02	0.04 ± 0.05		0.14 ± 0.10	0.01 ± 0.00	0.06 ± 0.00		0.02 ± 0.00	0.01 ± 0.00	0.33 ± 0.13	0.08 ± 0.01	0.05 ± 0.01	0.06 ± 0.01	0.10 ± 0.01
108	NI	2322	28.302									0.03 ± 0.00			0.02 ± 0.02				1.63 ± 1.14				
109	3,5-dimethoxy benzaldehyde	2336	28.412	0.03 ± 0.11		0.12 ± 0.03	0.05 ± 0.02	0.75 ± 0.13	0.66 ± 0.05	0.10 ± 0.03	0.05 ± 0.02	0.03 ± 0.03	0.03 ± 0.04	0.13 ± 0.03	0.04 ± 0.00	0.98 ± 0.41		0.18 ± 0.05	0.45 ± 0.02	0.56 ± 0.06	0.90 ± 0.12	0.06 ± 0.01	1.04 ± 0.19
110	NI	2342	28.459	0.06 ± 0.00		0.05 ± 0.02		0.01 ± 0.00	0.52 ± 0.03	0.06 ± 0.02	0.12 ± 0.05		0.05 ± 0.02		0.02 ± 0.01		0.09 ± 0.12	0.01 ± 0.00	0.09 ± 0.03	0.22 ± 0.35			
111	NI	2352	28.533	0.02 ± 0.00		0.02 ± 0.01	0.03 ± 0.01			0.03 ± 0.02			0.02 ± 0.00		0.03 ± 0.01		0.09 ± 0.11			0.07 ± 0.01			
112	chavicol	2394	28.83	0.08 ± 0.00	0.12 ± 0.05	0.09 ± 0.02	0.05 ± 0.01	0.05 ± 0.02	0.09 ± 0.01	0.14 ± 0.05	0.07 ± 0.01	0.05 ± 0.02	0.08 ± 0.05	0.05 ± 0.01	0.04 ± 0.02	0.24 ± 0.14		0.12 ± 0.05	0.5 ± 0.01		0.11 ± 0.03	0.05 ± 0.04	0.12 ± 0.06
113	NI	2411	28.958		0.04 ± 0.01		0.17 ± 0.04	0.08 ± 0.01	0.13 ± 0.01							0.05 ± 0.04	0.10 ± 0.01			0.07 ± 0.01	0.07 ± 0.02	0.02 ± 0.01	0.07 ± 0.01
114	NI	2419	29.012															0.02 ± 0.01					
115	NI	2442	29.173																		0.07 ± 0.01		0.04 ± 0.03
116	NI	2473	29.392			0.03 ± 0.01	0.03 ± 0.01									0.05 ± 0.03							0.04 ± 0.01
117	NI	2514	29.677				0.06 ± 0.01	0.02 ± 0.01		0.13 ± 0.01		0.07 ± 0.06			0.04 ± 0.00				0.06 ± 0.03				
118	NI	2518	29.707	0.03 ± 0.02		0.06 ± 0.02	0.03 ± 0.02		0.05 ± 0.01		0.04 ± 0.02					0.32 ± 0.30		0.05 ± 0.01		0.03 ± 0.03	0.07 ± 0.02	0.04 ± 0.01	0.04 ± 0.01
119	NI	2535	29.827	0.05 ± 0.01	0.10 ± 0.02	0.04 ± 0.02	0.05 ± 0.00	0.05 ± 0.02	0.05 ± 0.00	0.03 ± 0.03		0.02 ± 0.01	0.03 ± 0.01		0.05 ± 0.01		0.03 ± 0.00	0.06 ± 0.00	0.04 ± 0.01		0.03 ± 0.00		

120	NI	2557	29.973	0.03 ± 0.00	0.05 ± 0.00	0.05 ± 0.01	0.04 ± 0.01	0.02 ± 0.01	0.04 ± 0.00	0.06 ± 0.02	0.16 ± 0.05	0.04 ± 0.01	0.02 ± 0.01	0.02 ± 0.01	0.03 ± 0.00	0.03 ± 0.01	0.03 ± 0.01	0.03 ± 0.00	0.04 ± 0.02	0.03 ± 0.01		0.20 ± 0.04	0.03 ± 0.01
121	NI	2585	30.178	0.02 ± 0.01		0.04 ± 0.01	0.03 ± 0.01	0.03 ± 0.01		0.04 ± 0.00					0.01 ± 0.00		0.02 ± 0.00	0.03 ± 0.01					
122	NI	2596	30.262				0.08 ± 0.02										0.02 ± 0.00				0.03 ± 0.01		
123	NI	2607	30.342																	0.04 ± 0.01		0.04 ± 0.02	
124	NI	2609	30.358				0.05 ± 0.01									0.04 ± 0.02					0.04 ± 0.01		0.04 ± 0.01
125	NI	2654	30.686		0.17 ± 0.03	0.24 ± 0.02	0.06 ± 0.02	0.15 ± 0.04	0.23 ± 0.01	0.19 ± 0.04	0.04 ± 0.02	0.14 ± 0.02	0.14 ± 0.04	0.18 ± 0.04	0.16 ± 0.02	0.22 ± 0.09		0.15 ± 0.07	0.17 ± 0.01	0.17 ± 0.01	0.21 ± 0.04	0.02 ± 0.00	0.22 ± 0.08
126	NI	2677	30.87	0.02 ± 0.01		0.04 ± 0.01	0.83 ± 0.12	0.03 ± 0.02	0.04 ± 0.01	0.03 ± 0.04					0.03 ± 0.01	0.02 ± 0.00	0.12 ± 0.02	0.03 ± 0.01				0.04 ± 0.01	0.05 ± 0.01
127	NI	2733	31.349		0.04 ± 0.00		0.03 ± 0.01												0.07 ± 0.02				
128	NI	2747	31.461				0.05 ± 0.02									0.05 ± 0.01	0.02 ± 0.00	0.06 ± 0.02	0.04 ± 0.01				
129	NI	2795	31.928	0.04 ± 0.00	0.05 ± 0.01	0.06 ± 0.03	0.08 ± 0.02	0.04 ± 0.01	0.06 ± 0.01	0.05 ± 0.01		0.05 ± 0.01	0.04 ± 0.00	0.05 ± 0.01	0.03 ± 0.01			0.04 ± 0.01	0.03 ± 0.00	0.05 ± 0.02			0.05 ± 0.01
130	NI	2809	32.067			0.03 ± 0.01	0.04 ± 0.02			0.05 ± 0.05	0.11 ± 0.04				0.02 ± 0.01		0.04 ± 0.01						
131	hexadecanoic acid	2887	32.916	0.06 ± 0.01	0.12 ± 0.04	0.11 ± 0.03	0.05 ± 0.00	0.06 ± 0.01	0.07 ± 0.01	0.15 ± 0.10	0.06 ± 0.03	0.08 ± 0.02	0.10 ± 0.06	0.14 ± 0.06	0.06 ± 0.01	0.12 ± 0.06		0.08 ± 0.02	0.01 ± 0.00	0.07 ± 0.02	0.15 ± 0.06		0.10 ± 0.05
132	octadecanoic acid	3030	34.921	0.02 ± 0.65	0.06 ± 0.07	0.07 ± 0.01	0.04 ± 0.01			0.18 ± 0.15	0.13 ± 0.02	0.08 ± 0.06	0.11 ± 0.15	0.06 ± 0.01	0.04 ± 0.02	0.04 ± 0.01	0.08 ± 0.01	0.06 ± 0.02	0.02 ± 0.00	0.04 ± 0.02	0.16 ± 0.08		0.09 ± 0.03

NI – not identified. t_R – retention time. min – minutes. RI – retention indexes. * RI values were compared to literature (https:// pherobase.com).

Table S2. Total antioxidant capacity, TPC and TFC in SBH from Espírito Santo state (Brazil).

Honey code	CRAC (TE)	FRAP (μmol $FeSO_4$ g^{-1})	DPPH (μg Trolox g^{-1})	TPC (mg GAE 100 g^{-1})	TFC (mg QE 100 g^{-1})
PEMC 01	1.51±0.07 [begqyφχ]	58.66±8.36	509.908±50.342 [a]	10.63±2.73 [a]	2.79±0.17 [adg4δψΞ₤₮]
PEMC 02	1.29±0.05 [acfho]	61.29±7.44	525.852±21.845 [b]	9.96±3.18 [bd]	3.14±0.43 [behy5εω₥₯]
PEMC 03	1.82±0.01 [adir17κξψΊ₭₵]	53.90±2.41	517.600±45.877 [c]	9.86±2.41 [ce]	2.77±0.19 [cfi6ζΑΟ₦₧₱]
CAMC 01	2.25±0.18 [bcdjms28αζλπωΌΓ₮₶]	51.38±1.48	413.801±69.107	13.80±0.61 [i]	3.93±0.24 [abclorvzσΒ₲]
CAMC 02	2.19±0.05 [efkt39βημρΐΎΔ₯₷]	59.72±1.79	454.587±10.867 [e]	15.79±0.69 [abcfjo]	4.07±0.16 [defjmpsx1ντΓ₳]
CAMC 03	2.40±0.12 [ghilnu40γθνςϋΏΕ₰₸]	54.88±3.40	427.666±61.367	15.26±0.21 [degkp]	4.04±0.25 [ghiknqtw2ξυΔ₴]
CVMC 01	1.37±0.40 [jklpvό]	68.53±23.53	490.070±84.529 [f]	14.71±0.82 [hlq]	3.32±0.24 [jku37ηΕ₵]
CVMC 02	1.65±0.13 [mnx5σύΰ]	44.69±3.23 [a]	447.369±79.816 [g]	12.55±1.55 [m]	2.92±0.24 [lmn8ηθΖΠ₱₶]
CVMC 03	1.93±0.02 [opw6τώΆ₱₹]	45.96±1.89 [b]	480.510±28.531 [h]	13.33±0.43 [n]	3.02±0.04 [opq9ιΗΡ₩₷]
CVMQ 01	0.86±0.06 [qrstuvxwδιΖ₲]	49.38±2.12 [c]	483.840±26.329 [i]	8.82±2.72 [fghuz]	2.54±0.20 [rstuακοΘΣ₪₸]
CVMQ 02	0.99±0.13 [y123456εΗ₳₺]	56.60±1.26	522.836±33.89 [j]	6.21±0.21 [ijklmnrstvwy1]	2.79±0.15 [vxwβλΙΤ₫₹]
CVMQ 03	1.31±0.09 [7890]	44.44±2.34 [d]	406.090±70.474	9.07±0.84 [opqx2]	2.37±0.09 [yz123γμπΚΥ€₺]
FGMC 01	1.50±0.25 [αβγδευΈΒ]	66.56±4.01	366.576±25.558	13.08±0.64 [r]	4.33±0.04 [456ρφΛ₻]
FGMC 02	1.45±0.40 [ζηθικλμνΉ]	79.51±30.29 [abcde]	487.945±37.541 [k]	11.81±3.99 [s]	4.44±0.22 [δεζηθικλμςχ₡₼]
FGMC 03	1.15±0.25 [ξπρςστυ]	48.96±3.01 [e]	430.935±41.490	11.16±0.40	3.27±0.30 [νξοπρςΜ₽]
FGMQ 01	0.85±0.04 [φχψωϊϋόύώΈΗΘ₴₻]	59.87±6.30	493.288±20.576 [l]	12.04±0.21 [t]	2.99±0.10 [στυφχΝ₢₵]
DBMC 01	1.11±0.31 [ΊΌΎΏΐΑΒ]	65.24±2.86	477.135±107.106 [m]	14.79±1.11 [uvx]	5.06±0.22 [ψωΑΒΓΔΕΖΗΘΙΚΛΜΝ₣₭₿]
DBMC 02	1.60±0.21 [ΓΔΕΖΗΘ]	52.01±5.13	512.175±33.811 [n]	13.23±0.64 [w]	3.73±0.27 [ΞΟΠΡΣΤΥ₡₢₣₵]
DBMC 03	1.50±0.25 [₭₮₯₰₱₲₳₴]	54.55±6.48	445.187±95.682 [o]	13.05±0.70 [y]	3.89±0.16 [₤₥₦₱₩₪₫€₭ℓ]
VDIMC01	1.44±0.09 [₵₶₷₸₹₺₻]	51.78±5.55	260.588±82.331 [abcfghijklmno]	14.80±0.76 [z12]	6.05±0.40 [₮₹₺₻₯₧₱₲₳₴₵₶₷₸₹₺₻₼₽₾₿₵ℓ]

Similar letters or numbers indicate that the means are statistically different at $p < 0.05$ according to Tukey test.

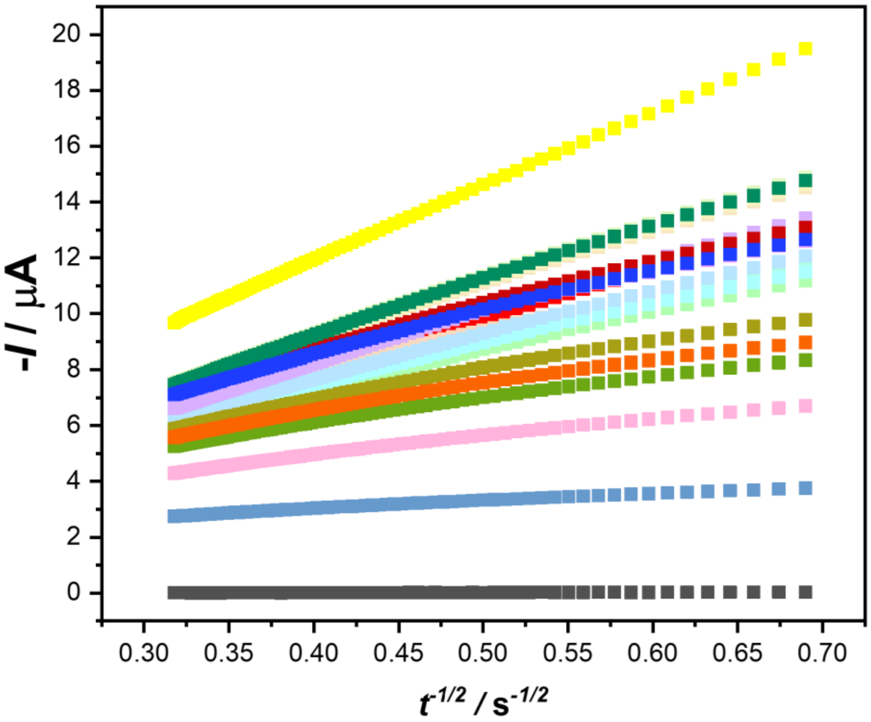

Fig. S1. Relationship between I and $t^{-1/2}$ from the Cottrell equation after reaction with 0.40 g mL^{-1} of honey solutions in 0.50 mol L^{-1} H_2SO_4. Legend: (■) *Melipona capixaba* CAMC 02; (■) *Melipona capixaba* PEMC 01; (■) *Melipona capixaba* CVMC 02; (■) *Melipona capixaba* FGMC 03; (■) Mandaçaia FGM 01; (■) *Melipona quadrifasciata* CVMQ 01; (■) *Melipona capixaba* DBMC 01; (■) *Melipona capixaba* VDIMC 01; (■) *Melipona capixaba* DBMC 02; (■) *Melipona capixaba* DBMC 03 ; (■) *Melipona capixaba* FGMC 01; (■) *Melipona capixaba* FGMC 02; (■) *Melipona capixaba* CVMC 01; (■) *Melipona Quadrifasciata* CVMQ 03; (■) *Melipona Quadrifasciata* CVMQ 02; (■) *Melipona capixaba* PEMC 02; (■) *Melipona capixaba* PEMC 03; (■) *Melipona capixaba* CAMC 01; (■) *Melipona capixaba* CVMC 03; (■) *Melipona capixaba* CAMC 03; (■) Trolox, (■) H_2SO_4 0.5 mol L^{-1}; (■) Ce^{4+} 1.0 × 10^{-3} mol L^{-1}.
